# PRO‐LDM: A Conditional Latent Diffusion Model for Protein Sequence Design and Functional Optimization

**DOI:** 10.1002/advs.202502723

**Published:** 2025-06-30

**Authors:** Sitao Zhang, Zixuan Jiang, Rundong Huang, Wenting Huang, Siyuan Peng, Shaoxun Mo, Letao Zhu, Peiheng Li, Ziyi Zhang, Emily Pan, Xi Chen, Yunfei Long, Qi Liang, Jin Tang, Renjing Xu, Rui Qing

**Affiliations:** ^1^ State Key Laboratory of Microbial Metabolism School of Life Sciences and Biotechnology Shanghai Jiao Tong University Shanghai 200240 China; ^2^ Function Hub The Hong Kong University of Science and Technology (Guangzhou) Guangdong 511453 China; ^3^ Department of Mathematics School of Computation Information and Technology Technical University of Munich 80333 Munich Germany; ^4^ Electronic and Information Engineering School of Electronic Information Engineering Beihang University Beijing 100191 China; ^5^ The Lawrenceville School Lawrenceville NJ 08648 USA; ^6^ XtalPi Inc. New Area Free Trade No. 1 Life Science and Technology Industrial Park Phase 1 No. 2‐1, Pudong District Shanghai 200120 China; ^7^ Research Center for Intelligent Computing Platforms Zhejiang Lab Hangzhou Zhejiang 311121 China

**Keywords:** conditional generation, functional optimization, latent diffusion models, protein sequence design

## Abstract

The diffusion model has grasped enormous attention in the computer vision field and emerged as a promising algorithm in protein design for precise structure and sequence generation. Here PRO‐LDM is introduced: a modular multi‐tasking framework combining design fidelity and computational efficiency, by integrating the diffusion model in latent space. The model learns biological representations at local and global levels, to design natural‐like species with enhanced diversity, or optimize protein properties and functions. Its modular nature also enables the integration with alternative pre‐trained encoders for enhanced generalization capability. Outlier design can be implemented by adjusting the classifier‐free guidance that enables PRO‐LDM to sample vastly different regions in the latent space. The approach is demonstrated in generating a novel green‐fluorescence‐protein variant with notably enhanced fluorescence in multiple working scenarios along with increased solubility and stability. The model provides a versatile tool to effectively extract physicochemical and evolutionary information in sequences for designing new proteins with optimized performances.

## Graphical abstract

PRO‐LDM is a modular latent diffusion framework that learns hierarchical biological representations to enable efficient and high‐fidelity protein design. PRO‐LDM can design species that resemble natural sequences with enhanced diversity or optimize protein functions. It demonstrates strong performance in designing functional GFP variants with enhanced fluorescence and stability.

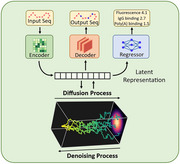

## 1 Introduction

Proteins are miniscule molecular machines that perform indispensable biological functions to sustain the life of organisms. Yet natural proteins only occupy a small fraction of the vast sequence space. Protein design probes into the unexplored territory, by modifying natural species or constructing sequences from scratch.^[^ ^1^ ^]^ Compared to rational design^[^ ^2^ ^]^ and directed‐evolution,^[^ ^1^ ^]^ computational design methods leverage the ever‐expanding protein databases to facilitate accurate sequence and structure generation, while reducing the reliance on high‐throughput experimental screening.^[^ ^3^ ^]^ Emerging deep learning‐based algorithms provided new computational toolkits that changed the paradigm in molecular biology research including protein structure prediction and protein design, which are problems on two sides of the same coin.^[^ ^4^ ^]^

Deep generative models are widely adopted due to their excellent track record in language and image processing. The state‐of‐the‐art (SOTA) generative model, i.e. diffusion model, can effectively sample complex distributions with integrative and controllable refinement processes that robustly generate high‐fidelity and more diverse data.^[^ ^5^, ^6^ ^]^ The current use of diffusion models in protein design primarily focuses on structure‐related tasks. Lee et al. developed a score‐based generative model ProteinSGM to design proteins with conformational folds not present in training sets, and generated structures that could insert masked sequences corresponding to native conformations.^[^ ^7^ ^]^ RFdiffusion from the Baker lab was derived from fine‐tuning the RoseTTAFold on protein structure denoising for main chain generation, which has been used for unconditional design, binder design, enzyme site design, etc.^[^ ^8^ ^]^ Other structure design frameworks such as FoldingDiff,^[^ ^9^ ^]^ DiffSBDD,^[^ ^10^ ^]^ DiffSDS,^[^ ^11^ ^]^ and Chroma^[^ ^12^ ^]^ also performed well in various tasks including single‐chain structure design, ligand docking, and protein complex generation.

However, high‐quality protein structural data is still lacking in terms of dataset size and granularity compared to sequence data. Sequence design is herein a more direct generative approach explored by researchers. Several groups reported the use of diffusion models in this approach, including EvoDiff, a framework that uses evolutionary‐scale data to generate natural‐like proteins,^[^ ^13^ ^]^ and LaMBO‐2, a method with diffusion‐optimized sampling to increase the yield and binding affinity of antibodies.^[^ ^14^ ^]^ However, these models were still limited by high computational demands during pre‐training with large datasets or evaluating the weights of amino acid positions, while demonstrations were mainly on sequence completion tasks and a few design cases. Such computational demands could be reduced by using a latent diffusion model. Similar pipelines were reported by integrating reinforcement or contrastive learning in the latent space, for fitness optimization or peptide ligand screening.^[^ ^15^, ^16^ ^]^ Herein, the capability of diffusion models to learn biophysicochemical properties within sequences for full‐length protein design remains to be explored.

On the other hand, the potential of an algorithm lies within its ability to address real biological problems beyond traditional engineering means, such as tuning the function of target proteins. Green fluorescent protein (GFP) is a β‐barrel protein with a chromophore center that fluoresces upon photonic excitation. The formation of an internal chromophore without external cofactors makes it an ideal fluorescent marker in a variety of scenarios, including labels for protein expression and localization, biosensors or cell markers, and indicators of protein–protein interactions and promoter activity.^[^ ^17^ ^]^ Enhancing the fluorescence intensity of GFP can increase the sensitivity, imaging resolution, and signal‐to‐noise ratio of the marker, thus further improving its utility.

Here we present PRO‐LDM (protein sequence generation with conditional latent diffusion models), a multi‐task modular learning framework that integrates a diffusion module in the latent space to achieve both design fidelity and model efficiency. PRO‐LDM is capable of extracting biological representations at both single amino acid and full sequence levels, as demonstrated by latent space visualization. Latent variable distributions are captured to generate meaningful embeddings for unconditional design of new sequences with native‐like properties and increased diversity. Global amino acid relationships are also reproduced. Conditional design produces new proteins within target fitness ranges, suitable for property and functional tuning. By adjusting the hyperparameter of classifier‐free guidance, PRO‐LDM can design outlier datapoints corresponding to species with properties and functions beyond those of natural proteins. We have demonstrated this approach by designing new GFP proteins with improved fluorescence, solubility, as well as chemical and thermal stabilities under a variety of working conditions, which further validated the efficacy of our algorithm.

The modular architecture of PRO‐LDM allows the integration of alternative pre‐trained models for improved scalability and adaptivity, as demonstrated by replacing the encoder with ESM2(8 M). Faster learning convergence and more discrete latent space mapping were achieved for the GFP task, while the generation of foldable sequences not associated with specific protein families was also achieved by training with more diverse datasets, such as CATH or Swissprot, to achieve dataset‐dependent *de novo* protein design. With advantages over single‐task algorithms in terms of reduced computational time and more effective performance, PRO‐LDM represents a modular, combinatorial new tool for efficiently extracting biological information from sequences and designing new proteins with distinct structures or target features that can be used in real‐world applications.

## 2 Results

### 2.1 The architecture of PRO‐LDM

**Figure 1:**
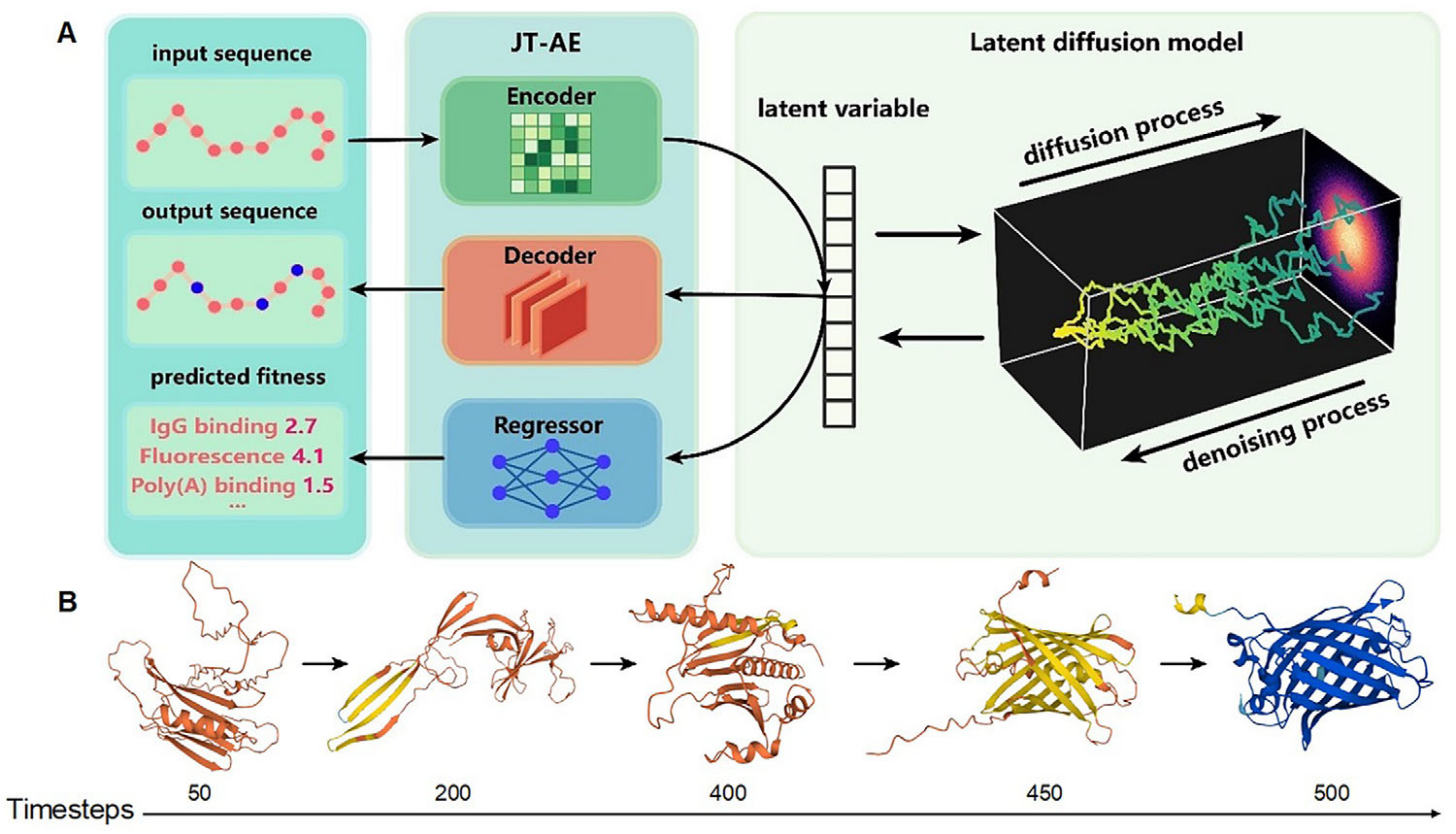
Overview of the PRO‐LDM architecture. A) In the training stage, input sequences are mapped into the latent space via a transformer‐based encoder. A latent diffusion model is applied to capture the distribution of the latent space. The latent variables are then used to reconstruct the sequence via a CNN‐based decoder and simultaneously predict the fitness via an MLP‐based regressor. In the sampling stage, the latent variables of new sequences are generated via the denoising process of LDM starting from a simple noise distribution. The output sequences and predicted fitness are obtained using the decoder and regressor, respectively. B) 3D‐structure iterations of a generated GFP during the denoising process colored to the pLDDT value (deep blue: pLDDT > 90; light blue: 90 > pLDDT > 70; yellow: 70 > pLDDT > 50; orange: pLDDT < 50). We select several time intervals in the denoising process trained on the GFP dataset and decode the latent variables into sequences. AlphaFold2 is used to predict the 3D structures.

PRO‐LDM is based on a jointly trained autoencoder (JT‐AE) with a conditional latent diffusion module to learn fundamental patterns embedded in protein sequences (**Figure** **1**). The model performs both unconditional and conditional protein sequence design with increased diversity. A fitness label is assigned to denote a particular property or function in a given set of proteins. When trained on sequence datasets with label values, PRO‐LDM can conditionally generate proteins toward a target label and predict their fitness simultaneously. When unlabeled datasets are used, or labels are uniformly set to 0, PRO‐LDM performs unsupervised learning to unconditionally generate proteins similar to the training sets.

#### 2.1.1 JT‐AE

JT‐AE is the fundamental structure of ReLSO^[^ ^18^ ^]^ and a combination of supervised and unsupervised learning in an autoencoder framework. It consists of a transformer‐based encoder, a convolutional neural network (CNN)‐based decoder, and a multilayer perceptron (MLP)‐based regressor in parallel. The output of the encoder is subjected to dimension reduction by a bottleneck module composed of fully connected layers, to project each sequence into a latent variable *z*. The collection of all *z* values constitutes the latent space. The latent variable *z*, together with the labels representing sequence fitness, are simultaneously passed to the diffusion module to simulate the distribution in the latent space, as described below.

#### 2.1.2 Conditional Latent Diffusion Model

Although capable of generating high‐fidelity data with different distributions, diffusion models are computationally expensive since the sampling process often requires thousands of network evaluations when applied directly at large spatial and temporal scales.^[^ ^19^ ^]^ To overcome this problem, our model adopts the diffusion process in the latent space to reduce the dimensionality of the input data, unlike other models that use diffusion all through the whole process. LDM learns the sequence data distribution in the latent space and captures its characteristics with different fitness. It employs a UNet as the neural network backbone and utilizes ancestral sampling for the generation process, such as denoising diffusion probabilistic models (DDPM).^[^ ^20^ ^]^

During the training phase, given an input sequence *x*, the encoder *f*_θ_ encodes *x* into a latent representation *z*  = *f*_θ_ (*x*), and the decoder *g*_θ_ reconstructs the sequence from the latent variable *z*, giving $\tilde{x}=g_{\theta }\;\left(z\right)=g_{\theta }\;\left(f_{\theta }\left(x\right)\right)$. In the latent space, we divide the diffusion process into *T* steps and add Gaussian noise according to a variance schedule $\beta_{1},\text{…},\beta_{T}$:

(1) $$\begin{array}{ccc} q\left(z_{1:T}|z_{0}\right) & = & \prod_{t=1}^{T}q\left(z_{t}|z_{t-1}\right),\;q\left(z_{t}|z_{t-1}\right)\\ & = & N\left(z_{t-1};\sqrt{1-\beta_{t}}z_{t-1},\;\beta_{t}I\right) \end{array}$$

where $z_{0}∼p\left(z_{0}\right)$. The reverse process could be defined as a Markov chain, starting at $p_{\theta }\;\left(z_{T}\right)=N\left(0,\;I\right)$:

(2) $$\begin{array}{ccc} p_{\theta }\left(z_{0:T}\right) & = & p\left(z_{T}\right)\prod_{t=1}^{T}p_{\theta }\left(z_{t-1}|z_{t}\right),\;p_{\theta }\left(z_{t-1}|z_{t}\right)\\ & = & N\left(z_{t-1};\mu_{\theta }\left(z_{t},\;t\right),\;\sum_{\theta }\left(z_{t},\;t\right)\right) \end{array}$$

β_*t*_ is kept constant as a hyperparameter, where $\alpha_{t}=1-\beta_{t},\;{\bar{\alpha }}_{t}=\prod_{s=1}^{T}\alpha_{s}$, $\sum_{\theta }\left(z_{t},\;t\right)=\sigma_{t}^{2}\;I$, and $\sigma_{t}^{2}={\tilde{\beta }}_{t}=\frac{1-{\bar{\alpha }}_{t-1}}{1-{\bar{\alpha }}_{t}}\;\beta_{t}$. Consequently, we sample $z_{t-1}∼p_{\theta }\left(z_{t-1}|z_{t}\right)$ using the following equation:

(3) $$\begin{array}{c} \;z_{t-1}=\frac{1}{\sqrt{\alpha_{t}}}\;\left(z_{t}-\frac{\beta_{t}}{\sqrt{1-{\bar{\alpha }}_{t}}}\varepsilon_{\theta }\left(z_{t},\;t\right)\right)+\sigma_{t}\gamma ,\;\;\;\gamma ∼N\left(0,\;1\right) \end{array}$$

where ε_θ_ is a function approximator designed to predict ε from *z_t_*. In the case of conditional sampling, the latent variable *z* is drawn along with class label *c*, so that the function approximator is changed to ε_θ_(*z_t_*, *t*,  *c*). We jointly train an unconditional diffusion model *p*_θ_(*z*) parameterized through a function approximator ε_θ_(*z_t_*, *t*), along with the conditional model *p*_θ_(*z*|*c*) parameterized through ε_θ_(*z_t_*, *t*,  *c*). During training, with a probability *p_uncond_*, the condition *c* is replaced with the unconditional class identifier ∅, enabling the model to learn both conditional and unconditional denoising within a single unified framework.^[^ ^21^ ^]^ The model then perform sampling using the following linear combination of the conditional and unconditional score estimate:

(4) $$\begin{array}{c} {\tilde{\varepsilon }}_{\theta }\;\left(z_{t},\;t,\;c\right)=\left(1+\omega \right)\;\varepsilon_{\theta }\left(z_{t},\;t,\;c\right)-\omega \varepsilon_{\theta }\left(z_{t},\;t\right)\; \end{array}$$

where ω is a hyperparameter controlling the strength of the classifier‐free guidance. Consequently, we train the latent diffusion model using the following equation:

(5) $$L_{LDM}=E_{\varepsilon \left(x\right),\;c,\;\varepsilon ∼N\left(0,\;1\right),\;t\;}\left[{\left\|\varepsilon -{\tilde{\varepsilon }}_{\theta }\left(z_{t},\;t,\;c\right)\right\|}_{2}^{2}\right]$$

This architecture can accelerate sequence generation speed and improve model efficiency. Moreover, we circumvent the need for classifier‐guided diffusion, which requires an additional pretrained classifier that may further increase model complexity. Instead, our classifier‐free guided diffusion combines conditional and unconditional diffusions for joint training, striking a balance between model complexity and computational costs, without reducing the quality in sequence generation, which exhibits notably superior efficiency compared to both ReLSO and other SOTA models (Table S1, Supporting Information).

The model is trained by minimizing the loss below:

(6) $$\begin{array}{ccc} L & = & \left\|g_{\theta }\left(f_{\theta }\left(x\right)\right)-x\right\|+\left\|h_{\theta }\left(f_{\theta }\left(x\right)\right)-y\right\|\\ & & +E_{\varepsilon \left(x\right),\;c,\;\varepsilon ∼N\left(0,\;1\right),\;t\;}\left[{\left\|\varepsilon -{\tilde{\varepsilon }}_{\theta }\left(z_{t},\;t,\;c\right)\right\|}_{2}^{2}\right] \end{array}$$

The first and second terms gauge the loss of JT‐AE, where *f*_θ_ represents the encoder, *g*_θ_ represents the decoder, *h*_θ_ represents the regressor, *x* is the input sequence and *y* is the corresponding fitness. The third term measures the loss of the latent diffusion model which is described above in detail.

### 2.2 PRO‐LDM Learns Representations of Protein Sequences

Our model was first trained unconditionally on protein datasets without fitness values, to evaluate the capture of inherent property or function representations in sequences. PRO‐LDM was able to design new protein variants by learning information embedded solely in sequences. The model was trained on each of three datasets, including two datasets on homologs of bacterial luciferase obtained from InterPro (IPR011251), and one dataset on a family of bacterial MDH enzymes (EC1.1.1.37). The luciferase datasets were used in two different forms, namely, Luciferase_MSA and Luciferase_RAW, to determine how multiple sequence alignment (MSA) affected the model's learning efficiency and generative performance.

**Figure 2:**
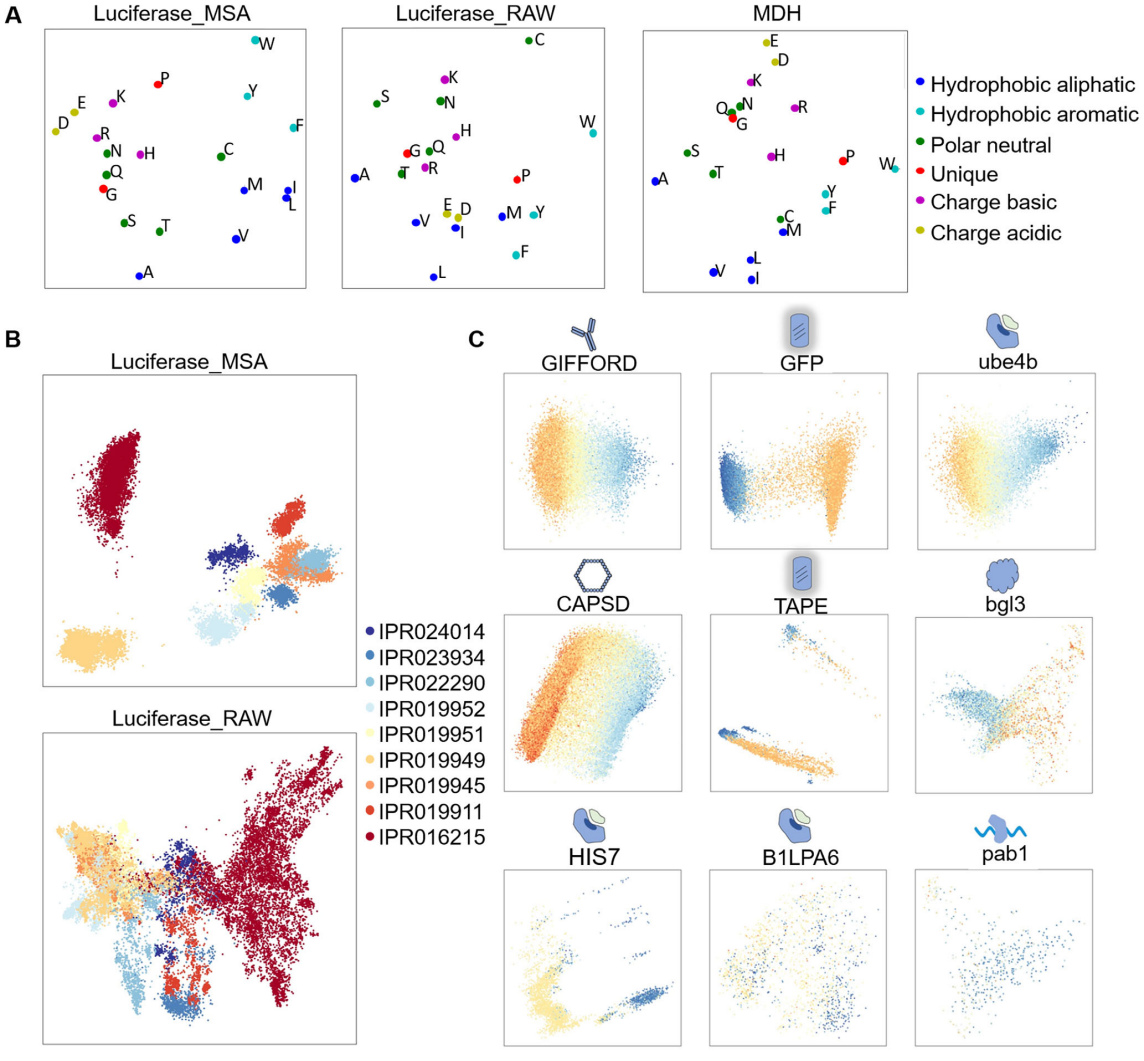
Protein representations at amino acid and sequence levels. A) Average latent space representations of amino acid characteristics learned by PRO‐LDM. (dataset from left to right: Luciferase_MSA, Luciferase_RAW, MDH) B) Organization of latent space reflecting subfamilies of Luciferase. Visualizations illustrate the latent representation of sequences in Luciferase_MSA (top) and Luciferase_RAW (bottom), project into the first two principal components and are colored by sub‐family annotations derived from InterPro. Only sequences belonging to the nine largest subfamilies are shown. C) Latent space representations of labeled protein sequences. The latent embeddings of nine labeled datasets learned by PRO‐LDM are displayed. The protein sequence representations are visualized by PCA, and each point is colored according to its corresponding fitness value. From blue to orange: high fitness to low fitness.

The propensity and distribution of 20 amino acids in a given sequence define the structure and function of proteins. It is therefore essential for a learning algorithm to capture the intricate biochemical attributes of amino acids. Amino acids with similar side‐chain structures and physicochemical properties are likely to be more correlated during the learning process than those that are not. An example of this is shown in Figure S1 and Table S2 (Supporting Information) by extracting amino acid embeddings in randomly selected sequences. Analysis on whole datasets was processed using the Principal Component Analysis (PCA) dimension reduction algorithm to visualize in the 2D space (**Figure** **2**A), where amino acids more similar are positioned closer together, such as charged acidic and basic amino acids. In contrast, amino acids with different biochemical properties are spaced further apart, such as non‐polar and polar amino acids. The results indicated that our model was able to learn characteristics of amino acids solely from their appearances in sequences.

Using the luciferase dataset as an example, we evaluated the efficiency of our model in learning comprehensive representations of protein sequences. Luciferase proteins were classified into different subfamilies based on their fold, the information of which was extracted from InterPro, and nine largest subfamilies were used for analysis. Sequence embeddings and family information were visualized in Figure 2B, where results for both Luciferase_MSA and Luciferase_RAW are shown. Apparent clustering is observed for sequences belonging to the same subfamily in both training sets. The results clearly showed that our model captured not only characteristics of amino acids from their positional appearance within sequences, but also grasped attributes at full protein level in terms of properties and functions, which are prerequisites for subsequent design tasks.

Nine deep mutation scanning (DMS) datasets were used to train PRO‐LDM. These datasets contain mutant sequences with equal or unequal lengths, as well as both indels (insertions/deletions) and amino acid substitutions (Experimental Section; Table S3, Supporting Information). Dimension reduction of sequence embeddings was performed by PCA in 2D space, with fitness values represented by different colors. As shown in Figure 2C, latent space visualizations of most datasets exhibit a global organization of fitness, providing the basis for subsequent conditional protein design. PRO‐LDM also showed comparable accuracy in protein fitness prediction as JT‐AE in all nine datasets (Table S4).

### 2.3 PRO‐LDM Unconditionally Designs Sequences Resembling Natural Proteins

The core objective of a generative model is to produce new data with a similar distribution to the original data. PRO‐LDM was designed to perform this task in the absence of a fitness label. The performance of PRO‐LDM has also been compared to VAE (variational autoencoder) based models, as they are notable for the ability to capture embedded information that distinguishes protein sequences and generate native‐like sequences in the target latent space.^[^ ^22^ ^]^

**Figure 3:**
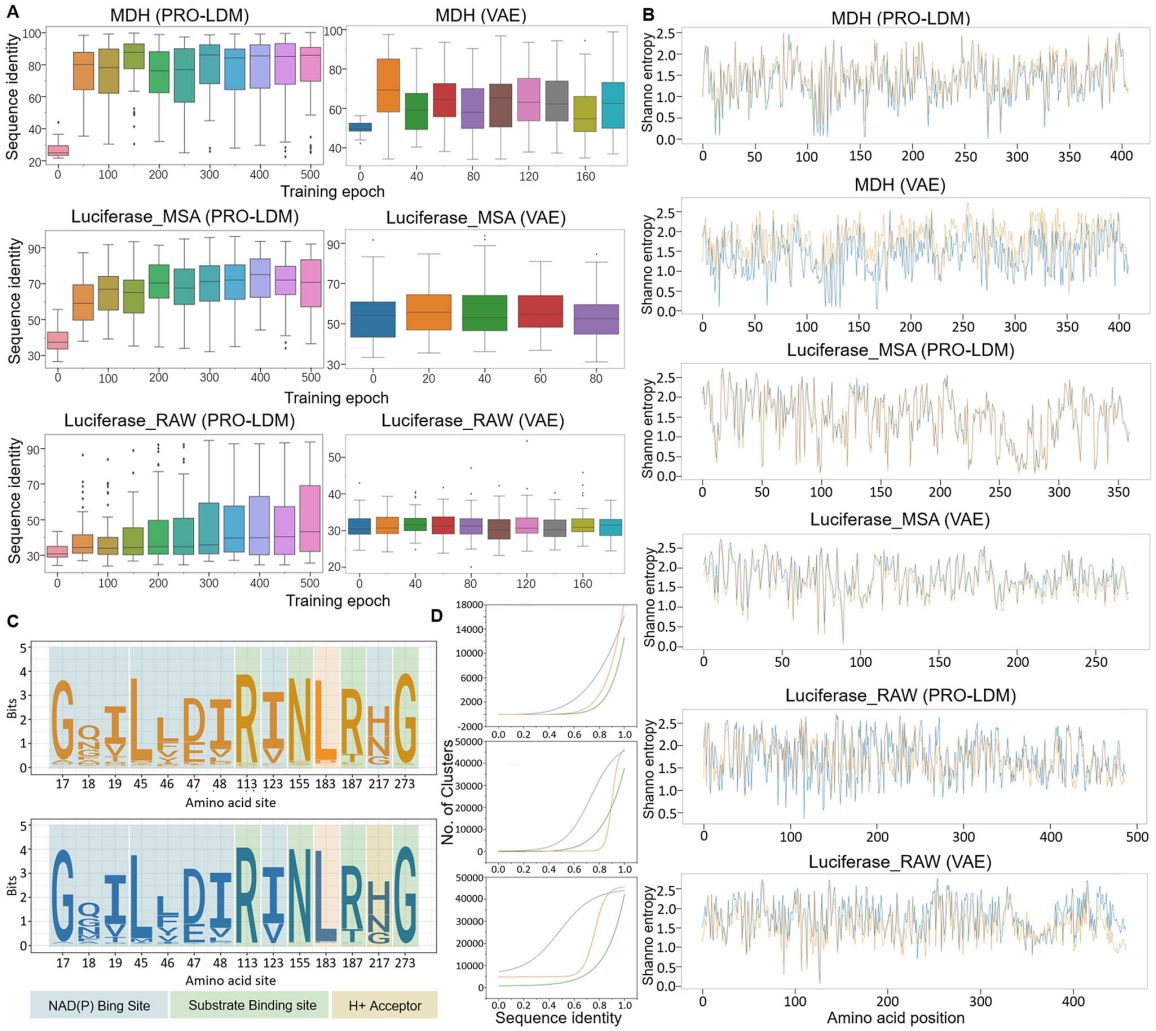
Unconditional protein design by PRO‐LDM. A) Sequence identity of generated sequences to the nearest natural sequence from training data at different iterations. X axis: training epoch; Y axis: sequence identity between generated sequence and best‐matching sequence in training set. B) Positional variability of PRO‐LDM or VAE generated sequences (orange) versus natural sequences (blue). X axis: amino acid position; Y axis: Shannon entropy. C) A sequence logo figure of key conserved function‐related positions in the MSA of MDH datasets (top: generated sequences; bottom: training sequences). D) Comparison of sequence diversity for natural and generated sequences in three datasets (top: MDH; mid: Luciferase_MSA; down: Luciferase_RAW; blue: PRO‐LDM generated sequences; orange: VAE generated sequences; green: training sequences).

The progress of training was monitored by comparing the identity between generated and natural sequences through calculating the proportions of identical residues in both sets. Sixty‐four sequences were generated in every 50 epochs. The identity between generated and natural sequences were observed to increase along with training steps (**Figure** **3**A, left). For the MDH dataset, PRO‐LDM resulted in higher median identities than VAE at the same epochs and achieved a higher level of convergence (Figure 3A, top left vs. right). For the luciferase_MSA dataset, the identity of PRO‐LDM was lower than VAE in the first 50 epochs, but reached a higher final convergence level (Figure 3A, middle left vs. right). For the luciferase_RAW dataset, the VAE model showed poor performance with a consistently low level of identification (the highest value was less than 40%). In contrast, PRO‐LDM achieved a final identity of 90% or higher, showing significantly improved learning capability (Figure 3A, bottom left vs. right). Comparing two luciferase datasets, we found that training PRO‐LDM with MSA data led to faster convergence and generated sequences more similar to natural proteins. Thus, integrating evolutionary information during training can further enhance the learning efficiency of the algorithm.

Amino acid conservations in proteins are associated with critical structural and functional motifs due to nature's selection process.^[^ ^23^, ^24^ ^]^ Such positional variability in sequences can be determined by calculating the Shannon entropy, for each site in the MSAs of generated and training sets.^[^ ^25^, ^26^ ^]^ Sequences generated by PRO‐LDM exhibited highly similar Shannon entropy profiles compared to those in the training sets (Figure 3B; Figure S2 and Table S5, Supporting Information), indicating that critical residue positions and evolutionary conservation patterns from natural sequences were reproduced. In the MDH dataset, PRO‐LDM demonstrated remarkably superior performance over comparisons, which reduced the positional mean entropy error (m.s.e.) of VAE and JT‐AE generated sequences by ≈11.2‐ and ≈13.7‐fold, respectively. In two luciferase datasets, the positional variabilities for PRO‐LDM generated sequences and natural sequences exhibited a high degree of similarity (with an overall correlation coefficient greater than 0.76), and the m.s.e. was slightly superior to that of the VAE and JT‐AE models (Figure 3B; Figure S2 and Table S5, Supporting Information). In addition, the high similarity of Shannon entropy between generated and natural proteins in MDH and luciferase_RAW inferred the capture of intrinsic evolutionary patterns even without information from sequence alignments.

Enzymes in the MDH dataset need to bind both the substrate and NAD^+^ cofactor to carry out catalytic functions. Therefore, we predicted functional sites of generated sequences using InterPro and marked them in the logo figure. Highly similar and conserved patterns were observed for the predicted amino acid occupations at respective positions between the training and generated sets (Figure 3C). Together with Shannon entropy profiles, these results demonstrated that PRO‐LDM was able to identify and utilize key evolutionary information in proteins to design new sequences resembling natural species with key positions and residues retained, in terms of protein scaffold and function.

Both amino acid composition and 3‐D conformation contribute to the functionality of proteins. Key residues responsible for the same function might be spatially adjacent in the folded protein, but being far apart in the primary sequence. Since the sequence‐based model has linear inputs, we wondered whether the global relationship in distant residue pairs could be grasped by our algorithm. Frequency distributions were calculated for each amino acid pair at all positions across sequences within MSAs. The correlation of frequency distributions was determined in both training and generated sets. PRO‐LDM showed very similar pairwise relationships to natural sets (Figure S3, Supporting Information) and outperformed the VAE model with higher average correlations (Table S6, Supporting Information). We also investigated whether generated sequences retained key functional domains reported in previous studies. Ten sequences from the generated set of MDH were randomly selected and examined for the presence of two key domains (“Ldh_1_N”and “Ldh_1_C”) as in the Pfam database, each containing more than 100 amino acids and were far apart from each other in the primary sequence.^[^ ^27^ ^]^ Both domains appeared in 9 out of 10 sequences, and only the “Ldh_1_C” domain appeared in the sequence “random_generated_4.” The results suggest that PRO‐LDM can design new variants preserving long‐distance amino acid relationships and key functional domains as in natural proteins (Figure S4, Supporting Information).

Across three datasets tested above, the diversity of sequences generated by PRO‐LDM significantly exceeded that of both VAE generated and natural sets at the same level of sequence identity within the cluster (Figure 3D). For instance, in the case of MDH design, the diversity of PRO‐LDM designed sequences exceeded that of the VAE model and natural data by up to two‐fold at 85% identity. We then assessed the in vivo stability of designed proteins using the sequence order of amino acids, estimated by the biopython instability index, where a value below 40 indicates high stability.^[^ ^28^ ^]^ The stability of PRO‐LDM‐generated sequences was found to be similar to training sets for MDH and Luciferase_MSA (Figure S5, Supporting Information) with instability index universally lower than 40. Yet generated sequences were less stable than the natural set in luciferase_RAW, which may be attributed to a higher sequence diversity and length variety. Finally, we compared distributions of amino acid types for generated and natural sequences (Figure S6, Supporting Information), which showed high agreements in all three datasets. Herein, we demonstrated that PRO‐LDM can unconditionally design protein sequences with higher diversity than the training datasets, while maintaining the stability, evolutionary and physicochemical characteristics that define proteins’ native structures and functions.

To compare the performance of PRO‐LDM with alternative diffusion‐based models, we benchmarked it against EvoDiff. The model captures evolutionary patterns in sequences by conditioning on MSA data of related proteins, which can be used to guide the design process, such as generating a query sequence harboring embedded features of the dataset.^[^ ^13^ ^]^ Here we compare PRO‐LDM's performance in designing a foldable protein with MSA information. Using the luciferase‐MSA dataset, PRO‐LDM was trained to generate 1 000 new sequences. For EvoDiff, we first utilized the model with pretrained checkpoint to generate query sequences conditioned on 64 MSA sequences, using either maximum or random subsampling. Moreover, we also fine‐tuned and trained EvoDiff from scratch on the Luciferase_MSA dataset, followed by unconditional sequence generation. Two sequences with highest pLDDT from EvoDiff were compared with the three longest sequences with fewest padding characters generated by PRO‐LDM. Structures predicted by AlphaFold3 are shown in Figure S7 (Supporting Information). Sequences generated by PRO‐LDM showed significantly better foldability, as indicated by higher pLDDT (per‐residue Local Distance Difference Test), compared to those generated by EvoDiff, demonstrating the usability of our model for MSA‐based unconditional design.

The modularity of the PRO‐LDM framework was further demonstrated by creating a PRO‐LDM(ESM2) version model through replacing our original encoder with that in ESM2, which carried transferable weights from pre‐training on the UniProt database that can enhance the model's generalization capability. Such modularity makes PRO‐LDM more robust in feature extraction and precision protein design, especially when pre‐trained on a universal dataset with sequence and structure diversity. PRO‐LDM(ESM2) was trained on either Swissprot or CATH datasets. The former features expert‐curated functional proteins in nature while the latter is a dataset based on protein structure classification. The trained model then generated *de novo* sequences not associated with specific protein families. AlphaFold3 predicted structures revealed decent foldability and primarily component of either β‐sheets (i) or α‐helices (ii‐iv) (Figure S8A, Supporting Information).

### 2.4 PRO‐LDM Designs New Proteins with Tailored Functional Properties

Despite indispensable roles in living organisms, the use of natural proteins ex vivo in therapeutic and biomedical applications are often limited by their natural properties or functional performances.^[^ ^29^ ^]^ Tuning native proteins on stability, solubility or enzymic activities are important aspects of protein engineering with enormous practical potential^[^ ^29^, ^30^ ^]^ We then explored whether datasets with fitness labels and conditional diffusion module could enable PRO‐LDM to design new protein variants with tailored properties or functions.

**Figure 4:**
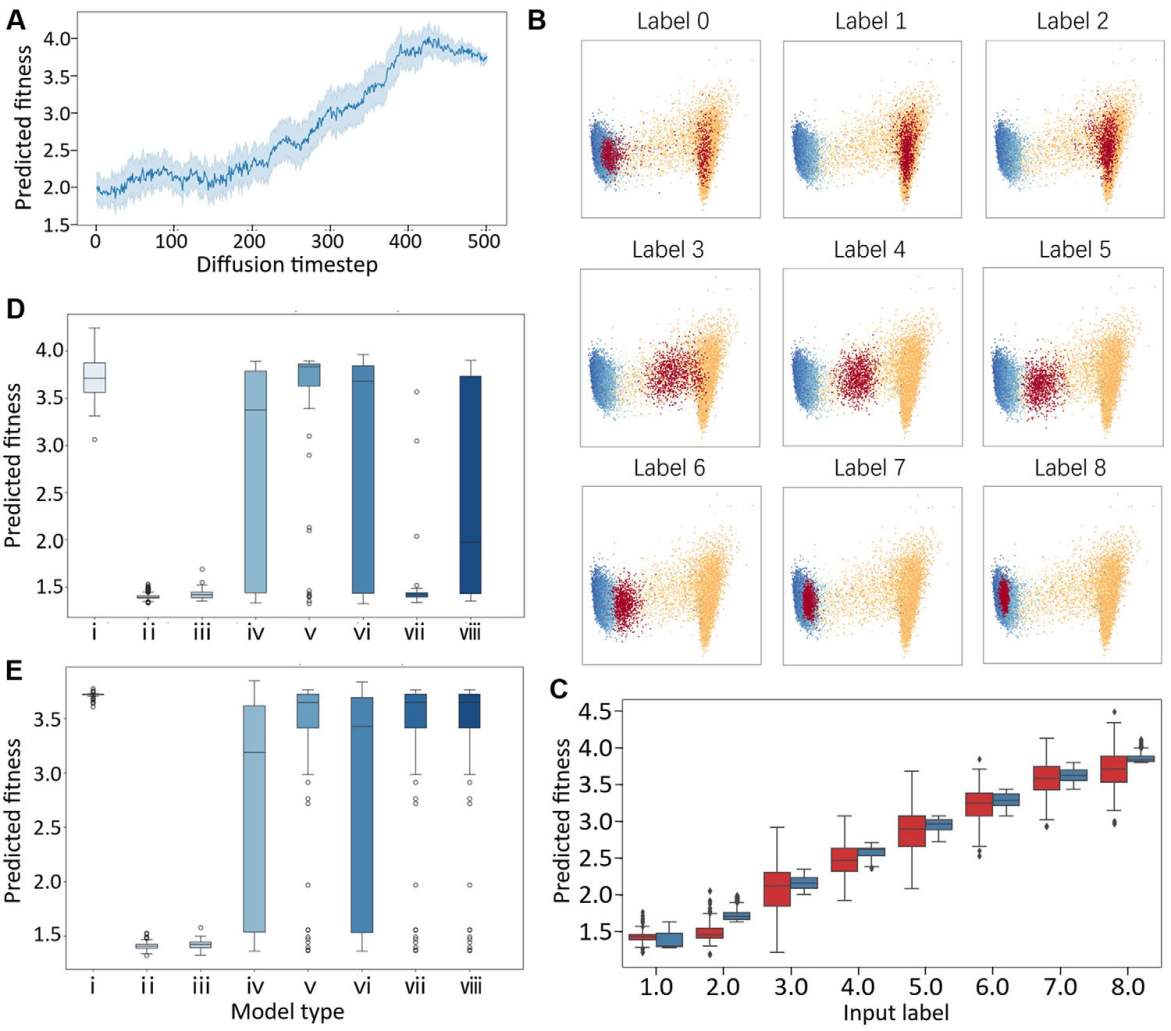
Conditional protein design by PRO‐LDM. A) The change of GFP predicted fitness in conditional protein sequence generation. To visualize the convergence of protein fitness into the targeted area, high‐fitness protein sequences are conditionally generated. The protein fitness is predicted using latent variables generated during the denoising process, utilizing the pre‐trained regressor. Sixty‐four protein sequences are generated for each dataset. The intermediate dark blue line represents the average fitness value. X axis: time step; Y axis: fitness. B) Natural sequences of the GFP dataset and conditionally generated protein sequences visualized in the latent space. The sequences in the GFP dataset are divided into eight labels based on their fitness, and new sequences are conditionally generated for each label. The generated (red) and natural sequences (cool‐color: higher fitness; warm‐color: lower fitness) of each label are mapped into the latent space and visualized using PCA. C) Fitness distribution of natural and generated sequences for GFP dataset in each label. Sixty‐four sequences of the GFP dataset are generated for each label, and their fitness is predicted using the regressor. The fitness distributions of generated sequences (red) are compared to that of natural sequences (blue). X axis: label; Y axis: fitness. D,E) PRO‐LDM (D) and ProteinBERT (E) predicted fitness comparison of generated sequences. Sixty‐four GFP sequences are generated from each model for comparison. X axis: model name; Y axis: predicted fitness. Model architecture for X axis: i) PRO‐LDM; ii) ProteinMPNN; iii) EvoDiff‐oadm‐38 M (training from scratch); iv) EvoDiff‐oadm‐38 M (finetuning); v) EvoDiff‐D3PM‐BLOSUM‐38 M (training from scratch); vi) EvoDiff‐D3PM‐BLOSUM‐38 M (finetuning); vii) EvoDiff‐D3PM‐uniform‐38 M (training from scratch); viii) EvoDiff‐D3PM‐uniform‐38 M (finetuning).

In our case, designing proteins with superior performance can be achieved by generating sequences with higher fitness values. The process was monitored by plotting changes in the sequence fitness over iterations. Across all nine labeled datasets, the sequence fitness progressively approached the target fitness values and converged through the denoising process (**Figure** **4**A; Figure S9, Supporting Information). This observation reflects the sampling principle of the diffusion model, which initiates random Gaussian noises and then gradually removes them until a target distribution is achieved (Figure 1).

Protein variants with different levels of a specific property were obtained by altering the input labels. When the label was set to 0, new sequences were unconditionally generated with a distribution resembling the training set in the visualization of latent vectors, similar to designs from unlabeled datasets in the previous section. When a value was assigned to the label, generated sequences showed clear alignment in their latent vector distribution against those with the same label in the training set (Figure 4B, Figures S10–S17). The fitness values predicted by the regressor for generated sequences varied along with input labels and exhibited similarity to corresponding sequences in the training data (Figure 4C; Figure S18, Supporting Information), suggesting that PRO‐LDM was able to design new variants with tailored properties or functional performance. The latent diffusion module refined the controllability on such fitness tuning, where the ReLSO architecture without diffusion generated undesired low fitness outlier sequences with notable functional discrepancies (Figures S19 and Figure S20, Supporting Information). A thorough discussion on performance comparison between PRO‐LDM and ReLSO without the latent diffusion is included in the supplement materials.

The generation capability of PRO‐LDM for functional proteins was benchmarked with SOTA models, including ProteinMPNN, EvoDiff, ESM3 and ProGen2, in the GFP design case, given the high quality of this dataset and its widespread use for model validation in various works.^[^ ^31^, ^32^, ^33^, ^34^, ^35^ ^]^ For EvoDiff, the model was trained either from scratch or fine‐tuned with the dataset. For ProteinMPNN and ESM3, pro_H variant with the highest fitness in the dataset was inputted as the initial structure for sequence decoding. For ProGen2, the model was also fine‐tuned with the dataset. To evaluate the similarity of amino acid distributions in generated and training protein sequences, we calculated the Reconstruction KL (Recon KL) for 1000 generated sequences against 1000 randomly selected test sequences.^[^ ^13^ ^]^ PRO‐LDM exhibited a significantly lower Recon KL compared to all models except ProGen2, indicating a closer resemblance to natural amino acid distributions. The minimum Hamming distance between generated and natural sets was calculated to assess the sequence diversity, where PRO‐LDM designed sequences showed lowest value (Table S7, Supporting Information).

In silico verification of generated GFP sequences was conducted from both structural and functional aspects. Both PRO‐LDM regressor and ProteinBERT were used to predict sequence fitness values, where proteins designed by PRO‐LDM exhibited higher functional predictions on both platforms compared to other models, suggesting better targeted optimization (Figure 4D,E; Figure S21, Supporting Information). The foldability of generated sequences was evaluated through structure prediction with Alphafold3 and reported by the average pLDDT. Except for EvoDiff‐oadm‐38 M (from scratch), designed sequences achieved average pLDDT scores exceeding 90, indicating reasonable foldability (Table S7, Supporting Information).

The benefit of model modularity was further evaluated by training GFP datasets with PRO‐LDM(ESM2). The model with a pre‐trained ESM2 encoder exhibited faster convergence during training and achieved comparable final performance in loss and sequence reconstruction accuracy to the original model (Figure S8B, Supporting Information), indicating a more robust feature extraction capability that integrates effectively with PRO‐LDM architecture. Dimensionality reduction to visualize protein sequences in relation with functions revealed higher mapping accuracy and datapoint separation to their labels (Figure S8C, Supporting Information). The generalization capability of PRO‐LDM(ESM2) in downstream tasks was verified by higher Pearson and Spearman correlations, as well as lower m.s.e. and L1 error of the regressor during training, indicating a stronger fitness prediction capability for PRO‐LDM(ESM2) (Figure S8D, Supporting Information). Such modularity endows our architecture with more flexibility for feature extraction and precision protein design in alternative use cases. Building on these results, one can perform conditional generation within a well‐structured latent space by leveraging a pretrained encoder and decoder with fixed parameters, as demonstrated by PLAID.^[^ ^36^ ^]^ The impact of using diffusion module based on the fixed, pretrained protein language model encoders was then evaluated. We employed ESM2 (8 M, 150 M, 3B) and ESM C (300 and 600 M) as encoders to visualize the latent representations of both training and generated data via dimensionality reduction, in order to compare diffusion behaviors across different latent spaces. Considering that latent vectors of different dimensions retain varying levels of sequence information, we also investigated the impact from latent dimensionality on the diffusion‐based generation process. For ESM C, increasing the parameter size from 300 to 600 M did not result in noticeable differences in the 2D distribution of latent variables. However, increasing the latent dimensionality led to earlier convergence, greater concentration of fitness among generated sequences, but also higher sequence redundancy (Figure S22A–F, Supporting Information). For ESM2, larger encoder sizes and latent dimensions both resulted in more dispersed latent distributions in 2D space. Generated sequences resided more concentrated in high‐fitness regions with increased redundancy (Figure S22G–K, Supporting Information). However, compared to models with frozen encoder weights, jointly training the encoder with the diffusion model yielded clearer mappings between latent variables and protein functions, which is more suitable for the GFP optimization task we conducted (Figure S22L, Supporting Information).

### 2.5 Outlier Design by Adjusting Classifier‐Free Guidance

Beyond generating new proteins in‐distribution with those from training sets, we also attempted to design significantly different variants by sampling outlier datapoints in the latent space. This method was used in out‐of‐distribution image generation and improved the generalization performance of ID tasks.^[^ ^37^ ^]^ New small molecules with enhanced properties in multiple domains were also designed using an out‐of‐distribution controlled diffusion model.^[^ ^38^ ^]^ We referred to the classifier‐free diffusion guidance in image generation and outlier sampling to elucidate the relation and boundary between sample diversity and fitness distribution in the directed‐generation task. The method was then demonstrated on the optimization of GFP for enhanced fluorescent intensity due to its relevance in biological applications such as live cell imaging.

In classifier‐free diffusion guidance, the sampling process is a linear combination of conditional and unconditional scores, as shown by Equation 4.^[^ ^21^ ^]^ The guidance strength is defined by a hyperparameter ω, which consequently controls the diversity and fidelity of generated samples.^[^ ^21^ ^]^ We herein evaluated the impact of ω on generated samples in the range between 0.1–1000. Our algorithm was benchmarked with SOTA models by cross‐referencing fitness prediction results on generated sequences with ProteinBERT^[^ ^39^ ^]^ (a self‐supervised deep learning language model for protein sequences), and Tranception^[^ ^40^ ^]^ (a transformer‐based fitness prediction model leveraging autoregressive predictions and retrieval of homologous sequences at inference).

**Figure 5:**
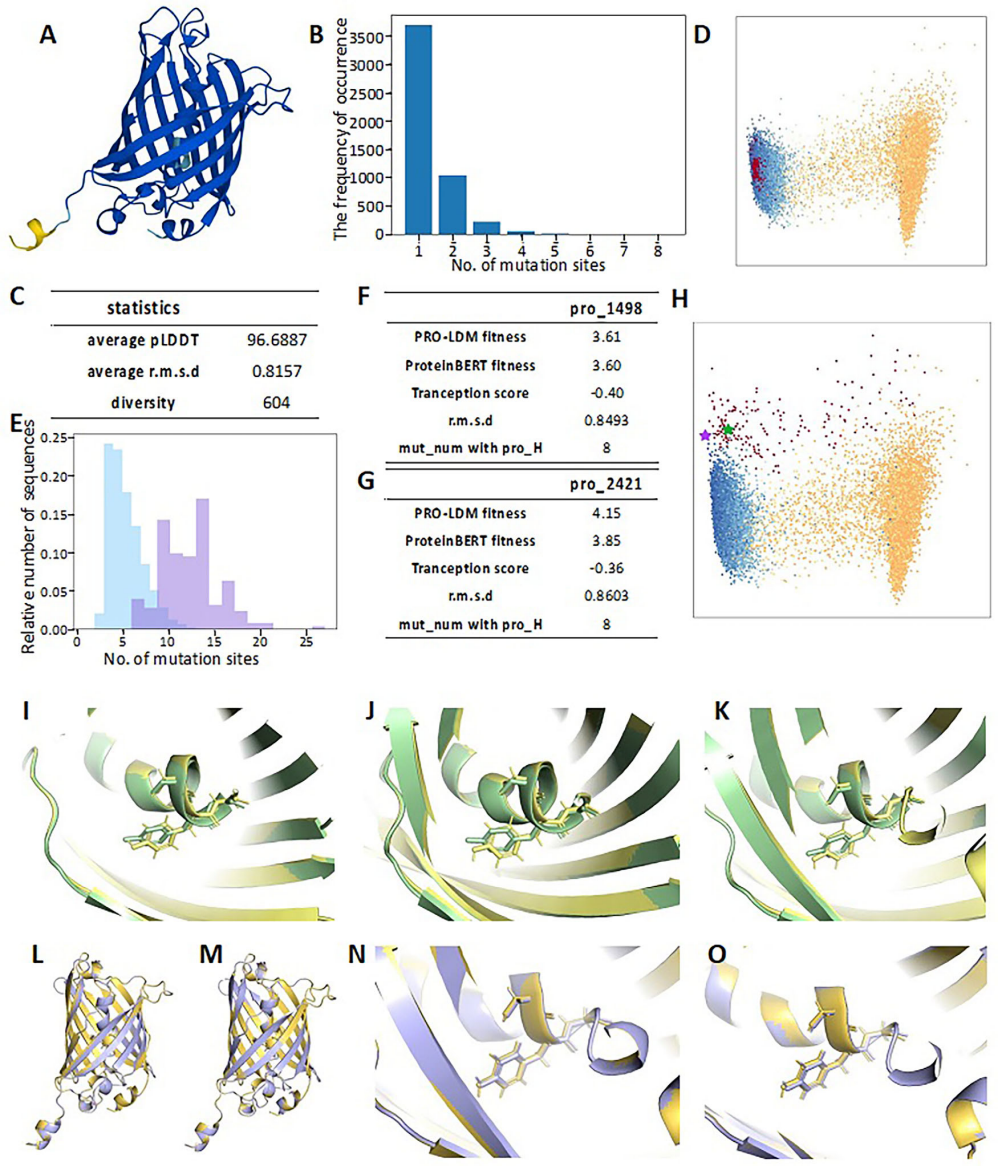
Outlier protein design by PRO‐LDM at ω = 1 and ω = 20 for GFP. A) Characteristic structure for designed GFP protein predicted by AlphaFold2 and colored to pLDDT at ω = 1. B) The number of mutation sites for 5000 generated sequences. X axis: number of mutation sites; Y axis: the frequency of occurrence. C) Statistical data of 5000 generated sequences at ω = 1. One hundred sequences are randomly selected to calculate the average pLDDT and r.m.s.d. compared with the protein with highest fitness in the training set (pro_H). D) Visualization of the latent space. The generated (red) and natural sequences (cool‐color: higher fitness; warm‐color: lower fitness) of each label are mapped into latent space and visualized by PCA. E) Histogram of mutation site counts for GFP training set and generated outlier samples when at ω = 20. X axis: number of mutation sites; Y axis: relative number of sequences (blue: training set; purple: generated set). F,G) Statistical data of pro_1498 and pro_2421 against pro_H. H) Visualization of the latent space for training set and the generated outlier sequences at ω = 20 (green star: pro_1498, purple star: pro_2421). I–O) Superimpositions of Alphafold2 predicted structures. I–K): local visualization of three key residues composing chromophore between wt‐GFP and I: pro_H, J: pro_1498, and K: pro_2421. L,M): Superimposition of pro_H with pro_1498 (L) and pro_2421 (M). N,O): Local visualization of three key residues composing the chromophore of pro_H against (N): pro_1498, and (O): pro_2421.

Decreasing the strength of classifier‐free guidance in diffusion model within a certain range can enhance the diversity of generated data but tended to decrease fidelity.^[^ ^21^ ^]^ In our case, when ω was set between 0.1 and 1.0, designed proteins all properly folded according to AlphaFold2 predictions (**Figure** **5**A; Figures S23A and S24A, Supporting Information). Decreasing ω resulted in gradually increasing sequence diversity (Table S8), accompanied by convergence of predicted fitness toward the targeted value (Figure 5D; Figures S23,S24,S25, Supporting Information). Designs with precise fitness values can be obtained when ω was set in this range, with minor enhancement on design diversity at lower ω values.

In contrast, when ω was set above 1.0, the diversity of generated sequences increased with the ω value. Datapoints representing generated sequences gradually moved beyond the distribution of the training set (Figures S26D,S27D, Supporting Information), leading to a significant increase in sequence diversity (Table S8; Figures S26C and S27C, Supporting Information), albeit with a decrease in predicted fitness (Figures S26E–G and Figure S27E–G, Supporting Information). Alphafold2 was employed to predict structures for 100 randomly selected sequences out of 1000 total samples. The average pLDDT was 96.09 at ω = 20 (Figure S27C, Supporting Information), while 87 of 90 proteins had r.m.s.d. (root‐mean‐square deviation) ≤1Å against pro_H, suggesting high conformational agreement between generated and training sequences despite of notably increased diversity. Our random selection has assigned pro_H to the test set, but it has a higher fluorescent intensity compared to best‐performing protein in the training set (calculated fluorescence: 13182 vs 12882). Herein, pro_H was selected as the control for subsequent experimental tests of designed protein variants.

When ω exceeds 20, PRO‐LDM is capable of generating increasingly diverse variants, with relative sequence identities against wild‐type GFP falling below 50% (Figure S28, Supporting Information). Variants with 50 to 120 mutations were subjected to structural prediction using AlphaFold3, with several sequences exhibiting pLDDT above 70, and the highest variant reaching 85.90 (Figure S29). In addition, when using the model trained on CATH dataset to generate sequences with the guidance scale ω = 20, the minimum Hamming distance between 1000 generated sequences and the training dataset was 0.87 ± 0.017, which is higher than 0.83 reported by EvoDiff. These findings suggest that PRO‐LDM is capable of sampling vastly different sequence space even given a highly similar training sequence set, that can generate low‐similarity and structurally plausible variants, reflecting its broad generalization capability. However, under the circumstance of GFP design, most generated datapoints with low sequence identity migrated toward areas closer to low fitness region in latent space, with a substantial decrease in predicted fitness by both ProteinBERT and Tranception (Figure S30, Supporting Information). In our case, decreased fitness is not in favor of high fluorescence in GFP and thus was not pursued further, although which might be relevant in alternative property tuning tasks such as decreasing the solubility for proteins self‐assembly.^[^ ^41^ ^]^ When ω was set at 1000, generated sequences exhibited aberrant conformations away from the native protein and low stability, resulting in a significant decline in pLDDT scores (< 50) (Figure S31, Supporting Information).

### 2.6 PRO‐LDM Optimizes GFP Variant with Enhanced Fluorescent Intensity

PRO‐LDM regressor, ProteinBERT and Tranception predicted notably different fitness distributions for generated sequences, especially at high ω values (Figures S26E–G, S27E–G, and S30E–G, Supporting Information). The difference can be attributed to the training process, whereas PRO‐LDM was trained on DMS data; Tranception was pre‐trained on UniRef100; and ProteinBERT was first pre‐trained on UniRef90 and finetuned using the DMS dataset. We considered predictions from all three models when screening sequences for subsequent experimental characterizations. Sequences generated at ω = 20 were selected due to their balance between diversity and fidelity in this setting. Outlier sequences with high fitness (label 7 and 8 predicted by our regressor) were screened according to their distribution against the training set in latent space, resulting in 180 datapoints (Figure. 5H, red dots). Figure 5E shows the number of mutations in outlier sequences in comparison to the training set, in which 168 out of 180 sequences had r.m.s.d. < 2 Å against pro_H (Table S9, Supporting Information). Two highest‐rated sequences, namely pro_1498 and pro_2421, predicted by ProteinBERT and Tranception were selected, both of which had r.m.s.d. < 1 Å and 8 mutations compared to pro_H, as well as 6 mutations against wt‐GFP. The minimum mutation number between the designed sequences and their most similar proteins in the training set is 3 and 4, respectively (Table S10, Supporting Information). The sequences for all four tested variants are provided in Table S11 (Supporting Information). We also analyzed the distribution of mutation sites and mutation frequency per sequence in the training set (Figure S32, Supporting Information), which showed that mutations are broadly distributed and most sequences contain fewer than ten mutations.

The fluorescence of GFP is influenced by the chromophore and its surrounding structural environment.^[^ ^42^ ^]^ Therefore, we aligned predicted structures of pro_1498, pro_2421 and pro_H against that of wt‐GFP. Similar deviation angles were observed between the phenyl rings of Y65 in pro_H, pro_1498, and pro_2421 against the same residue in wt‐GFP (Figure 5I–K). Further superimposition between pro_1498 and pro_2421 against pro_H showed highly similar side‐chain alignments in their chromophores (Figure 5L–O), which suggested a common conformation for higher fluorescence from both generated sequences and experimentally verified mutants as compared to wt‐GFP. A closer inspection of the chromophore center revealed hydrogen bonds between the hydroxyl group of Y65 and surrounding residues on the β‐barrel (H147, T202), which is present in wt‐GFP but absent in pro_H, pro_1498, and pro_2421. This missing interaction may account for reduced structural rigidity and fewer constraints on conformational changes associated with fluorescence (Figure S33, Supporting Information). In the six mutations from pro_1498 and pro_2421 to wild‐type GFP, only L63 is adjacent to the chromophore and engages in the polar interaction with V60, a residue involved in the chromophore's hydrogen bond network. The remaining mutations do not directly interact with the chromophore but may play roles in the structural support for protein functions (Figure S34, Supporting Information).

**Figure 6:**
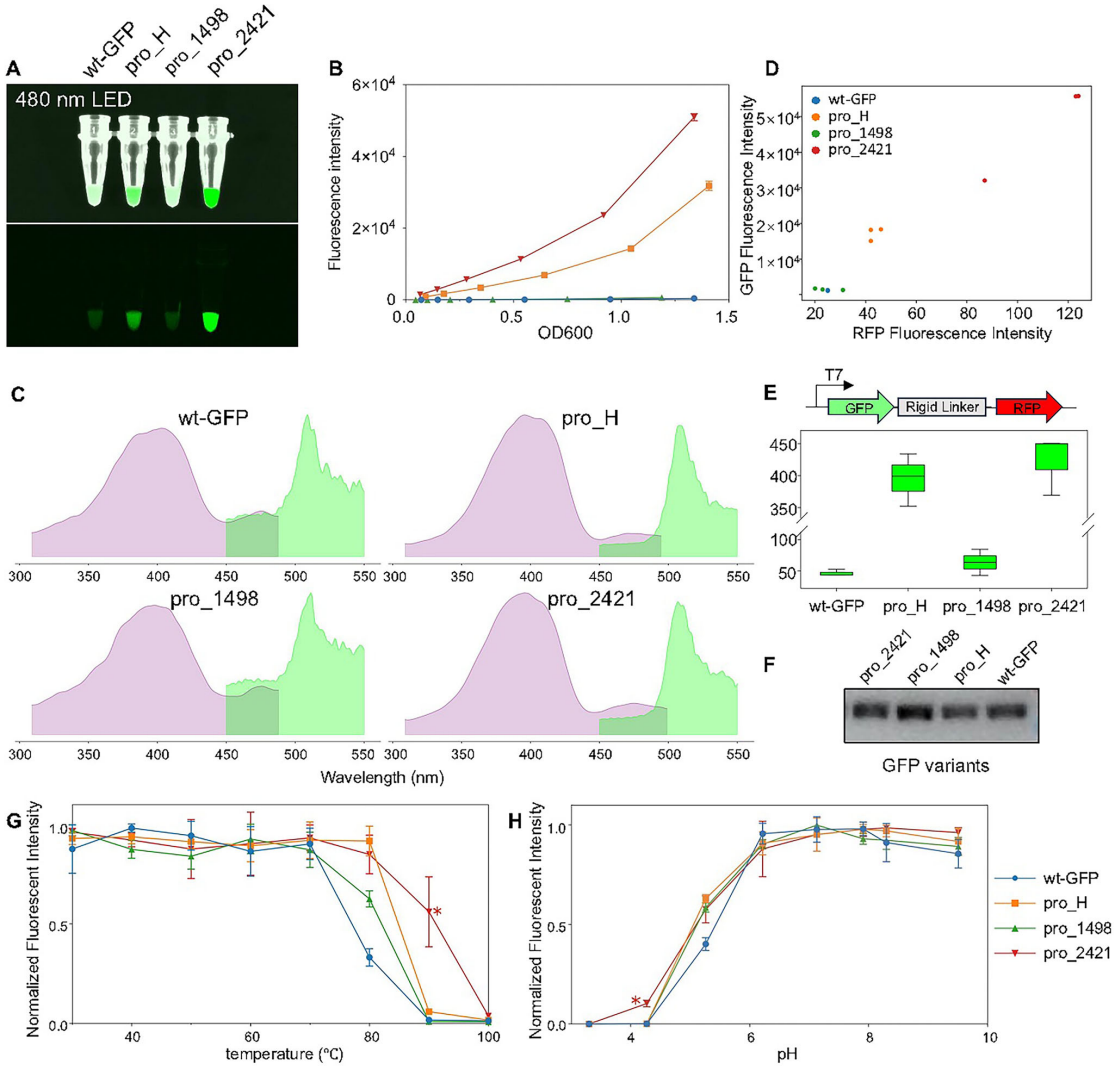
Experimental verification of PRO‐LDM designed GFP variants. A) Culture tubes of Rosetta (DE3) expressing four types of GFP under 480 nm LED without emission filters (left to right: wt‐GFP; pro_H; pro_1498; pro_2421). Bacterial cultures are adjusted to display almost identical optical density (OD600) by spectrophotometer. B) Fluorescence intensity of GFP variants as a function of concentration in bacterial culture (485 nm excitation filter and 520 nm emission filter). C) Fluorescence spectra of the four GFP variants. D) GFP against RFP fluorescence intensities in bacterial cultures from four variants (GFP: 395 nm excitation filter and 509 nm emission filter; RFP: 588 nm excitation filter and 633 nm emission filter). E) The box plot of green‐to‐red fluorescence intensities calculated from three repeat experiments (GFP: 395 nm excitation filter and 509 nm emission filter; RFP: 588 nm excitation filter and 633 nm emission filter). F) Western blot band for GFP‐RFP fusion proteins. G) The thermal stability of four GFP variants as determined by percentage fluorescence retained. H) The chemical stability of four GFP variants as determined by percentage fluorescence retained.

The two designed variants pro_1498 and pro_2421, together with wt‐GFP and pro_H, were expressed in Rosetta (DE3) for experimental assessment. All four GFP variants readily exhibited fluorescence in bacteria with pro_2421 showing highest brightness at the same bacterial density (OD600 = 1.0), under excitation at 485 nm (**Figure** **6**A). The fluorescent intensity of pro_2421 was 127.1‐fold higher than that of wt‐GFP, 58.7‐fold higher than that of pro_1498, and 2.1‐fold higher than that of pro_H (Figure 6B). Fluorescence spectrum scanning showed very similar profiles between wt‐GFP and pro_1498, as well as pro_H and pro_2421, respectively (Figure 6C). Maximum excitation wavelengths ranged from 395 to 405 nm and maximum emission wavelengths ranged from 505 to 515 nm, while pro_H and pro_2421 had more similar excitation and emission wavelengths (**Table** **1**).

**Table 1:** Summary of recent reports on GFP optimization.

Protein Name	Excitation Maximum [nm]^c)^	Emission Maximum [nm]^d)^	Molecular Brightness multiplier of [wt‐GFP]^e)^	Intracellular Brightness multiplier of (reference protein)^f)^			Expression (multiplier of wt‐GFP)^g)^	Mutation site number (reference protein)	Design Method	Ref.
				395–405nm	475–490nm					
EGFP	488	507	1.70	ND	1 (EGFP) ^a)^		ND	4 (wt‐GFP)	Rational design and screening	[44]
mGreen Lantern	503	514	3.71	ND	1.81(EGFP) ^a)^		ND	10 (Clover)	Rational design and screening	[45]
mNeon Green	508	522	4.70	ND	1.36 (EGFP) ^a)^		ND	16 (dLanYFP)	Computational design and directed‐evolution	[46]
StayGold	500	508	7.48	ND	1.85 (EGFP) ^a)^		ND	1 (CU17S)	Random mutagenesis and screening	[47]
StayGold‐ E138D	497	505	6.38	ND	0.76 (EGFP) ^a)^		ND	1 (StayGold)	Rational design	[48]
mStayGold	499	510	6.89	ND	1.85 (EGFP) ^a)^		ND	3 (QC2‐6)	Directed‐evolution	[49]
mBaoJin	500	508	6.02	ND	1.85 (EGFP) ^a)^		ND	10 (StayGold)	Directed‐evolution	[50]
AausFP1^h)^	504	510	8.34	ND	ND		ND	different species	Isolated form A. cf. *australis*	[51]
UniRep2^i)^	ND	ND	ND	<7.94 (wt‐GFP)			ND	<15 (wt‐GFP)^b)^	Deep learning	[32]
PROTLGN^j)^	ND	ND	ND	2 (wt‐GFP)			ND	1 (wt‐GFP)	Deep learning	[52]
wt‐GFP	405	509	1	1 (wt‐GFP)	1 (wt‐GFP)	1 (wt‐GFP, normalized to RFP)	1	0	This work	–
pro_H	395	508	1.38	8.51 (wt‐GFP)	60.83 (wt‐GFP)	6.84 (wt‐GFP, normalized to RFP)	0.73	4 (wt‐GFP)	This work	–
pro_1498	398	511	1.18	1.37 (wt‐GFP)	2.16 (wt‐GFP)	0.95 (wt‐GFP, normalized to RFP)	1.01	6 (wt‐GFP)	This work	–
pro_2421	396	507	1.66	9.12 (wt‐GFP)	127.14 (wt‐GFP)	6.69 (wt‐GFP, normalized to RFP)	0.83	6 (wt‐GFP)	This work	–

^a)^ estimate from figure 2f from^[^ ^48^ ^]^;

^b)^ random generation screened from a deep‐learning based prediction method;

^c)^ the excitation maximum of the state in nanometers. The excitation wavelength at which the fluorescence intensity is maximum, while keeping the emission wavelength fixed at 510 nm, during the scanning of excitation spectra;

^d)^ the excitation maximum of the state in nanometers. The emission wavelength at which the fluorescence intensity is maximum, while keeping the excitation wavelength fixed at 395 nm, during the scanning of emission spectra;

^e)^ molecular brightness is calculated as the product of the extinction coefficient and quantum Yield;

^f)^ intracellular brightness is calculated by normalizing the measurements using co‐expressed red fluorescent protein;

^g)^ expression is assessed through subsequent analysis using ImageJ after Western blot;

^h)^ the brightest GFP documented in FPbase;

^i)^ a RNN‐based deep learning model trained on UniRef50 and fine‐tuned sequences that are related to the protein of engineering interest;

^j)^ a structure‐based lightweight graph neural network designed to facilitate favorable mutants.

ND: No data

Additional experiments to normalize GFP fluorescent intensity against expression levels were carried out by fusing respective sequences to mKate2, a red fluorescent protein (RFP), using a rigid α‐helical linker, where green‐to‐red fluorescence ratios were determined. Two excitation wavelengths were used, i.e. 395 and 480 nm. Whilst 395 nm was the commonly used excitation wavelength for GFP performance evaluation, 480 nm was also widely utilized during live cell imaging applications due to the minimal cellular damage from visible light.^[^ ^43^ ^]^ Pro_2421 outperformed all other variants under 395 nm and showed slightly lower brightness at 480 nm against pro_H, despite its higher absolute fluorescent intensity in cell (Figure 6D,E and Table 1). The expression levels of fusion proteins were also examined by western blot and analyzed by ImageJ (Figure 6F). Interestingly, both pro_H and pro_2421 had lower expressions as compared to wt‐GFP in *E. coli* despite their enhanced fluorescent intensities (Table 1).

GFP variants were subjected to his‐tag purification for subsequent characterization of extinction coefficient (EC), quantum yield (QY), thermal stability and chemical stability (Figure 6G,H; Table S12, Supporting Information). Pro_2421 exhibited highest EC and QY amongst all four variants, showing higher molecular brightness over both wt‐GFP (1.66×) and pro_H (1.20×) (Table 1). Although all variants reported similar pKa values, pro_2421 exhibited superior tolerance in highly acidic condition, which still retained 10% of its maximum fluorescence at pH 4.27 when other proteins were fully denatured (Figure 6H). The variant also sustained higher temperatures, while 50% of its maximum fluorescence was retained after heating at 90 °C for 10 min (Figure 6G). In contrast, the brightness of other proteins dropped below 6% of their maximum. The enhanced performance of pro_2421 was likely to be related to its higher solubility and structural stability, since all pro_2421 proteins were expressed in the soluble fraction of *E. coli*, while other variants always showed fractions misfolded into insoluble pellets (Figure S35). Our designed variant pro_2421 has shown superior performance in multiple domains, demonstrating the feasibility of the outlier‐generating approach for protein property or functional optimization.

Table. 1 summarizes recent reports on GFP optimization from different groups.^[^ ^32^, ^44^, ^45^, ^46^, ^47^, ^48^, ^49^, ^50^, ^51^, ^52^ ^]^ Notable efforts from rational design, computational guided design and directed evolution, random mutagenesis and screening, and alternative deep learning based methods were included. Outlier generation with PRO‐LDM has demonstrated a feasible pathway by optimizing fluorescent intensity of the designed protein with a high increase ratio in multiple practical working scenarios,^[^ ^32^, ^50^, ^52^ ^]^ while simultaneously enhancing its biophysical properties including solubility, chemical, and thermal stability. Noteworthily, our method also performed the task in a *de novo* design‐based pathway by generating the sequence through training data‐guided denoising from random noise with a Gaussian distribution, rather than through a mutagenesis‐based route which focused on residues in the chromophore center (Figure S34, Supporting Information). This enabled to probe mutation sites distributed at various locations both proximal and distal to the chromophore, which again demonstrated our claim that PRO‐LDM was able to grasp and reproduce the global amino acid relationship in the earlier section. The approach enables more flexibility during design from both the reaction center and their surrounding scaffold that synergistically contribute to protein properties and functions, while showing more efficiency over random metagenesis and screening.^[^ ^32^, ^47^ ^]^

## 3 Conclusion

Our work introduces a deep‐learning framework striking at the sweet spot of design fidelity and computational efficiency by employing a diffusion module in the latent space. The model can effectively grasp embedded information in amino acids from their presence within protein sequences both locally and globally, to construct new proteins with enhanced diversity and tunable properties. Its modular architecture enables the reduction of computational power required for completing various tasks, without sacrificing the potential to integrate with pre‐trained large models. Diffusion model's capability to add noise to the input and reconstruct through denoising further improves the resilience and versatility of the encoder in the algorithm, producing diverse samples in an efficient manner. Compared to optimization algorithms such as ReLSO, PRO‐LDM does not require complex norm‐based negative sampling to achieve a convex latent space for gradient‐based optimization. The process is conveniently tunable using the classier‐free guidance hyperparameter ω, which allows outlier sequence generation to design novel protein variants that surpass the performance of native species, as demonstrated by the highly fluorescent and stable GFP reported in the manuscript. The wide distribution of mutated residues in both the chromophore center and surrounding scaffold further demonstrated the capability of PRO‐LDM to learn global representations from proteins sequences, while being versatile in reproducing them in a *de novo* rather than mutation‐based approach. When trained with more diverse data, the framework showed capability to generate protein species not associated with specific protein families in a dataset‐dependent manner.

PRO‐LDM has shown advantage over existing diffusion‐based sequence design models in several ways. EvoDiff features evolutionary‐scale sequence information for pre‐training to generate nature‐like proteins, including those with disordered regions.^[^ ^13^ ^]^ In comparison, PRO‐LDM doesn't require genome‐scale training data and is computationally efficient, which provides easy access for routine design work with optimization capability through outlier design that allows quick experimental verification in biolabs. LaMBO‐2 adopts a classifier‐guided diffusion modeling for sampling to extend the Bayesian optimization procedure for sequence design, which relies on the determination of positional weights in sequences via the gradient of the value function with respect to sequence embedding.^[^ ^14^ ^]^ Such process does not present in the transformer‐based PRO‐LDM, which learns contextual relationships between amino acids through a *de novo* approach. The elimination of the need to additionally evaluate the relative importance of different positions in specific properties or functions simplifies the design process.

Beyond the current stage of work, further improvements to the combination with structure‐based generative models may help to provide an end‐to‐end pipeline for highly precise protein scaffold customization, by building a parallel neural network that aligns information from both aspects. Such multimodal learning, integrated with publicly available and readily deployable large‐scale protein language models may also enable latent zero‐shot/few‐shot prediction and protein sequence generation.

Due to the compatibility of PRO‐LDM's directed‐design function with labeled sequence data, the model is not restricted to protein datasets. In principle, PRO‐LDM can also be trained on alternative sequence data such as genomic datasets, enabling its potential use in gene editing or RNA vaccine design. Such required annotated sequence datasets are being progressively generated from both high‐throughput omic technologies and computational prediction platforms, which may provide solid research and technological foundation for PRO‐LDM's application in a broader realm of molecular biology problems.

## 4 Experimental Section

### Network Architecture

The backbone of our model was a jointly trained autoencoder as developed in ReLSO. The encoder employed a four‐head transformer with six hidden layers, each having a dimension of 200. A bottleneck module, consisting of a fully connected layer, was applied to compress the embedded information into the latent space, projecting each sequence into a latent variable *z* with a default dimensionality of 64. The decoder used four 1D convolutional layers to reconstruct sequences from latent variables. Rectified linear unit's (ReLU) activation and batch normalization layers were incorporated between convolutional layers, except for the final layer.

In parallel with the decoder, an MLP‐based regressor, composed of one fully connected layer featuring a dropout rate of 0.2, was employed to predict fitness in our model. A classifier‐free diffusion guidance model was leveraged between the encoder and decoder, which consisted of a four‐layer 1D‐convolutional U‐Net that captured the disturbed latent distribution of each diffusion step, with the total number of diffusion steps set to 500 by default. This approach facilitated learning the latent space of the sequences and enabled the conditional generation of *z*.

### Network Training and Labeled Fitness

The distinct labels were assigned to sequences based on various fitness ranges. An 8‐label division method was adopted over rounding fitness values. The procedure preprocessed each dataset and visualized the relationship between dataset length and fitness distribution. Boundaries for fitness and sequence length were then established and uniformly divided into eight segments, with a few datasets being divided into five segments. For the unsupervised task, the labels were uniformly set to 0.

### Network Training and Sequence Generation

Sequences were fed into the encoder as strings during training, using an embedding layer with a dimension of 100, followed by the transformer learning the interdependencies between residues. A bottleneck layer then compressed the discrete high‐dimensional sequence information into a 64D latent variable *z*. All *z* values constituted a continuous, condensed latent space of sequences. The fitness label was simultaneously introduced into the conditional diffusion model as input to learn the distribution of information in the latent space. Finally, *z* served as input for both the decoder and regressor, where the fitness value was introduced during supervised learning for fitness prediction. The model was trained for 500 epochs using the AdamW optimizer with a cosine annealing learning rate starting at 0.00002 on four 32GB V100 graphics processing units and employing a batch size of 512.^[^ ^53^ ^]^ The diffusion timestep was set 500.

### Dataset Selection and Processing

Luciferase_RAW dataset: Hawkins‐Hooker *et al.* downloaded sequences containing a luciferase‐like domain (IPR011251) from InterPro (https://www.ebi.ac.uk/interpro/).^[^ ^22^ ^]^ The dataset contained 69,130 sequences with a maximum sequence length of 504 amino acids. The sequence identity threshold used during the splitting of the training and validation sets was 70%. The dataset was used to evaluate model's generation capability with training on homologous proteins within the same domain.

Luciferase_MSA dataset: Based on Luciferase_RAW, Hawkins‐Hooker et al. used Clustal Omega with the profile Hidden Markov Model (HMM) on the bacterial luciferase family from Pfam to create a MSA version of Luciferase_RAW dataset, incorporating additional evolutionary information for training.^[^ ^22^ ^]^

MDH dataset: Donatas et al. constructed the MDH dataset utilizing a family of bacterial malate dehydrogenase (MDH) enzymes.^[^ ^26^ ^]^ The dataset comprised 16,898 sequences, with an average length of 319 ± 18.2 amino acids. The pairwise identity of the sequences was based on a threshold of 10%. The identity threshold of sequence used during the splitting of the training and validation sets was set at 70%. MDH dataset was selected for dual validation of the unconditional generation capability of PRO‐LDM and evaluation of its generalization ability across diverse protein families.

Mutation datasets: We trained and tested conditional PRO‐LDM on nine deep mutational scanning datasets: Gifford,^[^ ^54^ ^]^ GFP,^[^ ^55^ ^]^ TAPE,^[^ ^56^ ^]^ Bgl3,^[^ ^57^ ^]^ Pab1,^[^ ^58^ ^]^ Ube4b,^[^ ^59^ ^]^ HIS7,^[^ ^60^ ^]^ CAPSD^[^ ^61^ ^]^ and B1LPA6.^[^ ^62^ ^]^ The effect of substitutions was evaluated using the initial six datasets, while the remaining three datasets were employed to examine the impact of insertions and deletions (indels). More details regarding these nine datasets could be found in Table S3 (Supporting Information).

Due to the unequal sequence lengths in the Luciferase_RAW, MDH and indels datasets, we employed padding symbols to align all sequences with the length of the longest sequence.

### Protein Structure Prediction by Alphafold2

Alphafold2 and Alphafold3 were used to predict structures for sequences generated during the denoising process.^[^ ^4^ ^]^ The service was provided for free by Zhejiang Gene Computation Platform (https://cloud.aigene.org.cn/) and Alphafold server (https://alphafoldserver.com/).

### Amino Acids’ Pearson's Correlation and Dimension Reduction

For each sequence randomly selected from dataset, a 2D matrix was generated to represent the likelihood of different amino acids occurring at each residue position in the matrix [20, seq_length]. Pearson's correlation was calculated to determine the relationship between each amino acid pair. The mean value was calculated from the amino acid embeddings of all sequences, followed by dimension reduction through PCA.

### Latent Space Representations of Protein Sequence Visualization

To evaluate the capacity of sequence‐level representation generated by PRO‐LDM in the latent space and to distinguish the functionality of proteins, the sub‐family accession numbers were obtained for luciferase from InterPro. The latent representation for the nine largest sub‐family proteins was encoded and generated. PCA was employed for dimension reduction and visualization of representations into 2D space.

### Identity Analysis of Generated and Natural Sequences

Sequences (number same as training set) were generated at 50‐epoch intervals for three unlabeled datasets (MDH, luciferase_MSA, luciferase_RAW) throughout the training process. BLAST (http://www.ncbi.nlm.nih.gov/BLAST/) was employed to conduct sequence alignments on generated sequences and calculate their identities in comparison to the natural set, represented in box plots.

### Multiple Sequence Alignments and Shannon Entropy

One thousand training sequences and 1 000 generated sequences were randomly selected, combined and input into Clustal Omega for alignment.^[^ ^63^ ^]^ In cases where mismatch occurred, non‐matching positions were replaced with a dash (“‐“), referred as a gap. The training and generated sequences were separated, and columns exhibiting over 75% gap ratio were removed. The Shannon entropies of both sets were calculated separately using the following equation:

(7) $$SE=-\sum_{i=1}^{20}p\left(x_{i}\right)log_{20}p\left(x_{i}\right)$$

where *p*(*x_i_*) represented the frequency of amino acid *i* in one column of the MSA.

### Logo Figure

One thousand sequences from both the training set and the generated set were selected and input into Clustal Omega for alignment.^[^ ^63^ ^]^ Frequency matrices were computed for both sequence groups, where conserved positions were identified. tBtool and R were employed for visualization. Both *ggseqlogo* ^[^ ^64^ ^]^ and *ggplot2* ^[^ ^65^ ^]^ in R were used to create and enhance the graphical presentations. The *gridExtra* package was used to consolidate the visualizations of both sequence groups and their correlation at the conserved positions.

### Pairwise Amino Acid Frequency Distribution

One thousand sequences were selected from both the training set and the generated set to perform multiple sequence alignment. Pairwise amino acid occurrence frequency matrices of dimensions [seq_length, seq_length] were computed for each sequence. The matrices were then reshaped to [1, seq_length × seq_length] and all sequences were concatenated in the training/generated set. Two groups of metrics were obtained with dimensions [seq_num, seq_length × seq_length], which were used to calculate the Pearson's correlation coefficient.

### Sequence Diversity Analysis

Generated sequences from PRO‐LDM and VAE models in alignments with the number of training sets were used. The MMseqs2 in MPI Bioinformatics Toolkit (https://toolkit.tuebingen.mpg.de/tools/mmseqs2) was used to cluster sequences at different identity threshold to obtain a diversity value, and Origin was used to fit the curves using non‐linear function.^[^ ^66^, ^67^, ^68^ ^]^

### Sequence Stability Analysis

Sixty‐four sequences were generated by PRO‐LDM and VAE models respectively, and compared with randomly selected 64 sequences from training sets. Biopython was used to calculate the instability index for each sequence, and matplotlib was used to make the box plot.^[^ ^28^ ^]^

### ProteinBERT and Tranception

ProteinBERT is a deep language model pretrained with Gene Ontology (GO) annotation predictions and tested with downstream tasks with diverse protein properties.^[^ ^39^ ^]^ The pretrained ProteinBERT was fine‐tuned using GFP dataset in TAPE^[^ ^31^ ^]^ before testing PRO‐LDM generated sequences. Tranception is a SOTA transformer‐based fitness prediction model employed to test PRO‐LDM generated sequences.^[^ ^40^ ^]^ A higher Tranception score indicated superior functionality.

### Average pLDDT and r.m.s.d

One hundred generated outlier sequences at different ω values were randomly selected for Alphafold2 structure prediction on Zhejiang Gene Computation Platform. We downloaded all successfully predicted PDB files, extracted the pLDDT value for each atom, and calculated the mean value for all atoms. The Superimposer module in *biopython* was used to calculate the r.m.s.d. value between the generated protein and pro_H.

### Superimposition of Predicted Structures

The predicted structures for the generated protein, pro_H, and wt‐GFP by Alphafold2 were used. Structure pairs were aligned in PyMOL (https://pymol.org) to visualize the superimposition of chromophores with hydrogen bond visualized.

### EvoDiff Training and Validation

The source code of EvoDiff (https://github.com/microsoft/evodiff) was downloaded for training on GFP dataset from scratch or fine tuning with 128 batch size. All three versions of the model (oadm38M, D3PM_BLOSUM_38 M, D3PM_UNIFORM_38 M) achieved convergence before the 10th epoch, and we used the 10th epoch checkpoint (both for training from scratch and fine tuning) to generate GFP sequences for analysis and comparison with PRO‐LDM.

The Recon KL was calculated using 1000 generated sequences and 1 000 randomly selected test sequences. The minimum Hamming distance was assessed between the 1 000 generated sequences and the entire training set. The r.m.s.d. against pro_H was calculated using the AlphaFold3‐predicted structures of five randomly generated GFP sequences (each model) and pro_H. Additionally, the sequence average pLDDT (seq avg. pLDDT) was derived from these AlphaFold3‐predicted structures. The minimum hamming distance, r.m.s.d. against pro_H and seq avg. pLDDT were reported for each model as the mean ± standard deviation.

For performance comparison of MSA sequences generation, the query sequences were generated from 64 luciferase_MSA sequences using random or max‐Hamming subsampled MSAs. In addition, we trained the oadm variant of EvoDiff from scratch and fine‐tuned it using the Luciferase_MSA dataset. The model trained from scratch reached convergence before the 70th epoch, while the fine‐tuned version converged before the 80th epoch. Accordingly, we used the checkpoints from the 70th and 80th epochs, respectively, for sequence generation. The sequence average pLDDT was also derived from the AlphaFold3‐predicted structures.

### Sequence Generation Using ESM‐3

We employed the webserver of EvolutionaryScale Forge to generate GFP variants. The server was configured with the esm3‐medium‐2024‐08 model. To guide the generation process, we used the AlphaFold3‐predicted structure of the pro_H protein as a structural prompt, and set the sampling temperature to 0.7.

### ProGen2 Finetuning and Sampling

The source code of ProGen2 (https://github.com/enijkamp/progen2) and the finetuning code (https://github.com/hugohrban/ProGen2‐finetuning) were downloaded. The GFP dataset was used to fine‐tune the ProGen2‐small model with a batch size of 32. The training loss converged prior to the 10th epoch, and the model checkpoint from the 10th epoch was subsequently used to generate novel GFP sequences. During sampling, the temperature was set to 1.0, and the prompt ‘<gfp>1SKGEELFTGV’ was provided to guide the generation of complete GFP sequences.

### PRO‐LDM(ESM2)

The ESM2 (8 M) model was used to replace the original PRO‐LDM encoder, with the tensor from its final representative layer fed into the pooling layer. The pretrained checkpoint was loaded and kept frozen during the first epoch, then unfrozen starting from the second epoch. Wandb was used to visualize the training process. For unconditional generation, PRO‐LDM(ESM2) was trained on Swissprot or CATH and the checkpoints at 50th epoch were loaded to generated new protein sequences.

### JT‐AE Ablation Study

The source code of ReLSO (https://github.com/KrishnaswamyLab/ReLSO‐Guided‐Generative‐Protein‐Design‐using‐Regularized‐Transformers) was dowloaded. For unconditional generation, the latent representation after training was sampled randomly and decoded to design novel sequences. For conditional generation, we followed the same optimization strategy reported in the article and source code.^[^ ^18^ ^]^ The fitness of generated sequences was evaluated using the regressor, and their latent representations were reduced in dimensionality using PCA. The visualization of box plots and scatter plots was accomplished using the Python package *Matplotlib*.

### TM Score

We used TMalign (https://zhanggroup.org/TM‐align/) to calculate the TM scores between predicted structures of generated sequences and training sequences, as well as the TM scores among the training sequences themselves.

### Protein Expression and Purification

The gene of wt‐GFP, pro_H, pro_1498 and pro_2421 were codon optimized, synthesized and cloned into pET‐28a (+) plasmid by GenScript. The plasmids were transformed into *E.coli* Rosetta (DE3) cells with kanamycin resistance. Single colonies were inoculated for seed culture at 37°C, 220 rpm for 16 h, which were transferred 1:100 to fresh LB medium. After OD600 values reached 0.6‐0.8, a final concentration of 1 mm IPTG (Isopropyl β‐D‐1‐thiogalactopyranoside) was added to induce protein expression. Cells were collected after 4 h by centrifugation for 3 min at 10 000 g, while cell pellets were collected and washed two times. Pellets were then resuspended in TBS buffer (0.05 M Tris‐HCl, 0.15 m NaCl) for subsequent characterizations. A rigid α‐helical linker GSLAEAAAKEAAAKEAAAKAAAAS was inserted between GFP and mKate2, to reduce intramolecular interactions and suppress Förster resonance energy transfer (FRET) between the fluorescent proteins.^[^ ^55^, ^69^ ^]^

For in vitro characterization, GFP‐6×His plasmids were generated for all four variants using the Golden Gate Cloning method. After expression, the cells were disrupted using ultrasonic homogenization (SCIENTZ‐IID), and the lysate was centrifuged (11627 × g, 10 min) to separate supernatant and pellet fractions. The fractions were then analyzed by SDS‐PAGE (MeilunGel). The proteins were purified from cell lysate through affinity chromatography using Ni Smart Beads 6FF (BDTL0063, Biodragon). The purified proteins were stored in 1×TBS (pH7.4) or 1× PBS (pH7.0) buffer.

### Fluorescent Intensity Characterization

1. Qualitative detection: Cell pellets resuspended in TBS buffer were adjusted to identical optical density by spectrophotometer and imaged at an excitation wavelength of 480 nm using ChemiScope 6200 (Clinx).
2. Quantitative detection of fluorescence intensity against OD600: Bacteria culture with GFP expressions were diluted by a factor of two from the highest concentration to six gradient concentrations. Samples were aliquoted into a 96‐well black plate with clear bottom, and a baseline of 50 mm TBS was established. The fluorescence intensities from each variant at 485 nm excitation wavelength were measured using the SPARK multimode microplate reader. The data were fit using the third order polynomial model. Three sets of replicate experiments were conducted.
3. Quantitative detection of fluorescence intensity against RFP: Bacteria culture with expressions of GFP‐RFP fusion proteins were diluted by 64‐fold, as the fluorescence intensity of pro_2421 exceeded the detection limit of the spectrophotometer at higher concentrations. The green fluorescence intensities at 485 nm excitation wavelength were measured using the Agilent BioTek Synergy H1 multimode reader. The red fluorescence intensities were measured at 588 nm excitation wavelength with 633 nm emission filter. Three sets of replicate experiments were conducted.

### Fluorescence Spectrum Scanning

Bacteria culture with GFP variant expressions were added to quartz cuvettes and a baseline of 50 mm TBS was established. The excitation spectra were obtained by scanning a range of excitation wavelength at 309–

490 nm with a fixed emission wavelength at 510 nm. The emission spectra were obtained by a range of emission wavelength at 450–550 nm with a fixed excitation wavelength at 395 nm. The fluorescence spectrum were acquired on FL 6500 Fluorescence Spectrophotometer.

### Western Blot

Bacteria lysates at the same OD600 reading were subjected to SDS‐Page gel‐electrophoresis on MeilunGel protein precast gel and transferred onto PVDF (polyvinylidene fluoride) membrane using iBlotTM3 Western Blot Transfer System. The transferred membrane was blocked with 4% skim milk (Nacalai Tesque, Inc.) in TBST (TBS with 0.05% Tween 20) for 1 h, and incubated with primary antibody (6 × His Tag Monoclonal Antibody, Invitrogen, 1:5000 dilution) overnight at 4 °C. The membrane was then washed 3 times with TBST buffer, and incubated with secondary antibody (Goat anti‐Mouse IgG (H+L) Secondary Antibody HRP, Invitrogen, 1:20000 dilution) for 1 h at room‐temperature. Highly sensitive plus ECL luminescence (Sangon Biotech) was used to visualize the proteins under Fusion FX Edge Spectra imaging system (Vilber Lourmat). ImageJ was used to compare sample intensities.

### Chemical Stability and pKa

The pH titration buffers were prepared in 50 mL centrifuge tubes and adjusted to pH = 3.14, 4.13, 5.16, 6.13, 7.09, 7.97, 9.28 and 10.27, respectively. The compositions for pH titration buffers are: 1) 100 mm citric acid/Na citrate (pH 3–5.5); 2) 100 mm KH_2_PO_4_/Na_2_HPO_4_ (pH 6–8); and 3) 100 mm NaOH/Glycine (pH 8.5–10). Each buffer (100 µL) was pipetted into the 96‐well plate in increasing incremental pH values. The first row was the control (pKa buffer and protein buffer) and the second row had 100 µL of GFPs added. After incubating at 30 °C for 60 min, the fluorescent intensity was measured using the Agilent BioTek Synergy H1 multimode reader. The chemical stability curves were plotted using Prism 10, and the pKa values were fitted and calculated by *CubicSpline* package in Python.

### Thermal Stability

GFP solutions (20 µL) in TBS (pH7.4) were added into 8‐tube PCR strips and heated from 30 to 90 °C with a 10 °C gradient, then maintaining the temperature for 10 min. The fluorescent intensity was measured using the Agilent BioTek Synergy H1 multimode reader.

### Extinction Coefficient

The EC was calculated by assuming that the peak value of NaOH‐denatured fluorescent proteins was the same as that of the NaOH‐denatured GFP‐type chromophore, which is 44 000 M^−1^ cm^−1^.^[^ ^70^ ^]^ The concentration obtained from the NaOH‐denatured sample was used to determine the peak extinction coefficient for the native sample. 0.125 m (final concentration) NaOH was used as the alkaline denaturant.^[^ ^70^ ^]^ The fluorescent intensity of denatured (measured at 447 nm) and native samples (measured at each protein's maximum excitation wavelength) was measured using the Agilent BioTek Synergy H1 multimode reader.

### Quantum Yield

GFP solutions in 1×PBS (pH7.0) were added to quartz cuvettes with the established baseline of 1×PBS. The quantum yield values were measured by Quantaurus‐QY Plus (HAMAMAYSU) at each protein's maximum excitation wavelength.

### Statistical Analysis

All statistical analysis was performed with the corresponding Python packages. Data presented in this work were expressed as the mean ± standard deviation (SD). The sample sizes used for statistical analyses differed across experiments. Specific numbers were provided in respective figures and detailed in respective sections, including *Multiple Sequence Alignments and Shannon Entropy*, *Logo Figure*, *Pairwise Amino Acid Frequency Distribution*, *Sequence Stability Analysis*, and *EvoDiff Training and Validation*. Box charts were determined by the 25th–75th percentiles. Mann‐Whitney U test was used to determine the statistical significance of observed differences between different amino acids with different properties and *p* < 0.05 was considered significant. Correlation analyses were conducted using both Pearson's correlation and Spearman's rank correlation, with the corresponding functions from the *script.stats* module utilized for computation.

## Conflict of Interest

The authors declare no conflict of interest.

## Supporting information



Supporting Information

## Data Availability

We have made the full codebase, datasets and checkpoints of PRO‐LDM publicly available on Github (https://github.com/AzusaXuan/PRO‐LDM/).
