# Supplementary material for: PRO‐LDM: A Conditional Latent Diffusion Model for Protein Sequence Design and Functional Optimization
Source: Adv Sci (Weinh). 2025 Jun 30;12(34):e02723. doi: 10.1002/advs.202502723 (PMC12442598; doi:10.1002/advs.202502723)
Supplement: Supplementary file 1 — Supporting Information [file ADVS-12-e02723-s001.docx]

Supporting Information

PRO-LDM: A Conditional Latent Diffusion Model for Protein Sequence Design and Functional Optimization

Sitao Zhang, Zixuan Jiang, Rundong Huang, Wenting Huang, Siyuan Peng, Shaoxun Mo, Letao Zhu, Peiheng Li, Ziyi Zhang, Emily Pan, Xi Chen, Yunfei Long, Qi Liang, Jin Tang, Renjing Xu*, Rui Qing*

**This Supporting Information includes:**

Supporting Discussion

Supplement Figures S1 to S35

Supplement Tables S1 to S12

SI References

**Supplement Discussion**

1. **Comparison between PRO-LDM and JT-AE**

JT-AE is the fundamental architecture of ReLSO, which jointly trains a transformer-based autoencoder with a fitness prediction network. ReLSO then added regularizations to the latent space, used labeled datasets to model the protein sequence-function landscape, and performed gradient ascent on fitness to converge into an optimum that can be decoded back into the sequence space. Ablation studies from three different perspectives were conducted to thoroughly evaluate the performance improvement obtained from adding a diffusion module: 1) unconditional generation on unlabeled datasets; 2) conditional generation on labeled datasets; and 3) computational resource requirements.

- 1. Unconditional generation comparison based on unlabeled datasets

For comparison of their unconditional generation capability, both PRO-LDM and JT-AE were trained on three unlabeled datasets (MDH, luciferase_RAW, luciferase_MSA) and sampled randomly in the latent space. Shannon entropy of generated sequences was superimposed with those from natural sequences, where significantly lower similarity was observed for JT-AE generated sequences (**Figure S2**, **Table S5**) compared to those from PRO-LDM (**Figure 3B, Table S5**). The results indicated more accurate sampling and capture of positional information for critical residue and evolutionary conservation patterns from natural sequences by the added latent diffusion module of PRO-LDM.

- 1. Conditional generation comparison based on the labelled datasets

The comparison between the conditional generation capability of PRO-LDM and ReLSO was conducted on the GFP functional optimization task. We evaluated all six sequence-space optimization methods in the iteration of ReLSO architecture, including MCMC (Metropolis-Hastings MCMC approach in latent space), MCMC-cycle (calibration of MCMC based on JT-AE), and MCMC-cycle-noP (the noise addition step has been removed from the MCMC-cycle), gradient-free optimization approaches such as hill climbing and stochastic hill climbing, and gradient ascent, the selected strategy for the final version of ReLSO algorithm. Sixty-four optimized latent representation were decoded into corresponding amino acid sequences and input into the regressor for fitness prediction (**Figure S19**). Fitness values for sequences generated by both MCMC and gradient ascent fell outside of the normal range and were unable to fold into functional GFP proteins. The remaining four strategies generated reasonable GFP sequences with notable outliers that far from the desired fitness range (Figure S19**, Figure S20, Figure 4C**). PRO-LDM had demonstrated superior control on sequence generation with well-tailored protein functions.

- 1. Computational resource requirement comparison

Last but not the least, PRO-LDM demonstrates superior efficiency compared to ReLSO in both training and sampling stages. PRO-LDM was trained for 5 hours on a single dataset over 500 epochs using four V100 GPU cards, while ReLSO required 6 hours and 21 minutes for 200 epochs on eight A800 graphic cards. During generation, PRO-LDM produced 64 samples within 3 minutes, whereas ReLSO generated 30 samples through each of six optimization methods (a total of 6×30 sequences) on CPU, taking a total of 3 hours and 50 minutes. A complete comparison of computing power requirement is presented in Table S1.

**Supplement Figures**

**
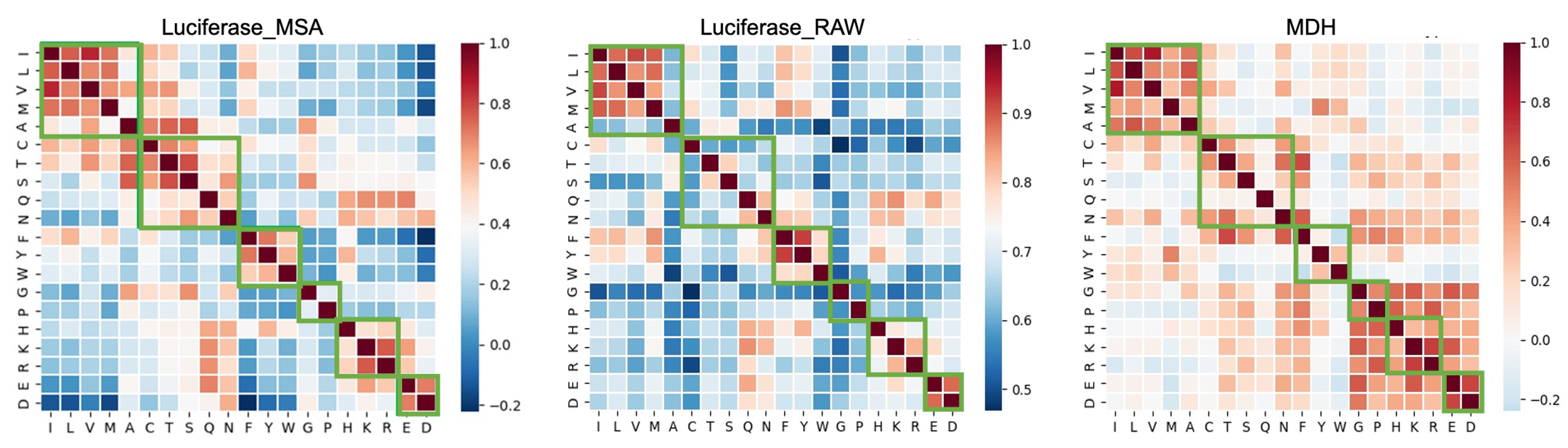
**

**Figure S1.** Pearson’s correlation between amino acid types in randomly selected sequences. For each output sequence, a 2D-matrix is generated to represent the likelihood of different amino acids occurring at each residue position. Pearson’s correlation is calculated to represent the relationship between amino acid pairs. Biochemically related amino acids exhibit higher correlation. Amino acid pairs within the same green border share similar characteristics. Dataset from left to right: Luciferase_MSA, Luciferase_RAW, MDH.

**Figure S2.** Positional variability of JT-AE generated sequences (orange) versus natural sequences (blue). **(A)** MDH; **(B)** luciferase_MSA; **(C)** luciferase_RAW. X axis: amino acid position; Y axis: Shannon entropy.

**
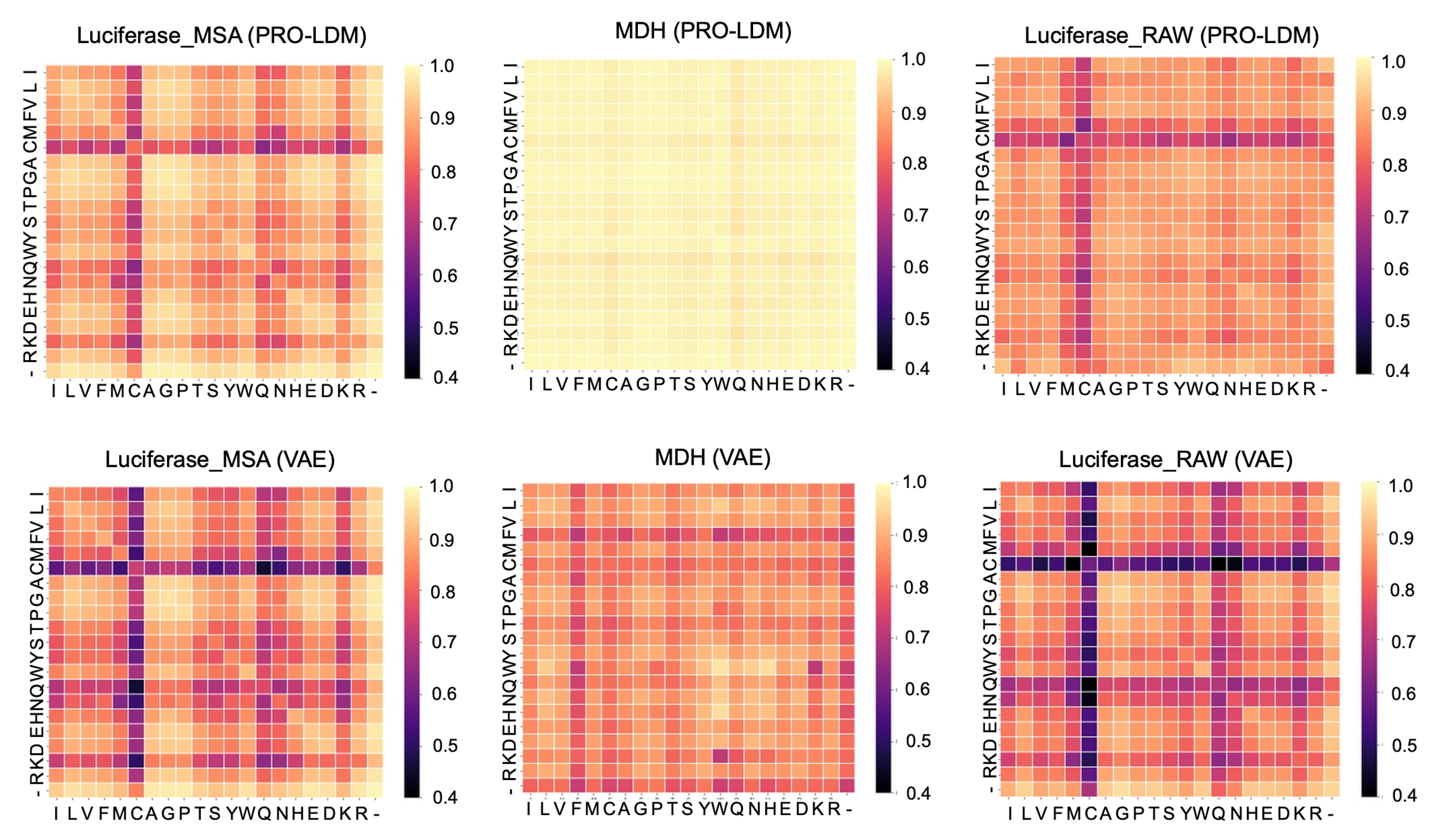
**

**Figure S3.** Amino acid pairwise correlations of generated and natural sequences. Each point on the map represents the correlation of amino acid pair frequencies between the MSA of natural sequences of Luciferase_MSA, Luciferase_RAW, MDH and those generated by PRO-LDM or VAEs. A high correlation denotes that the same pairwise long-distance interactions are found as in natural sequences.

**
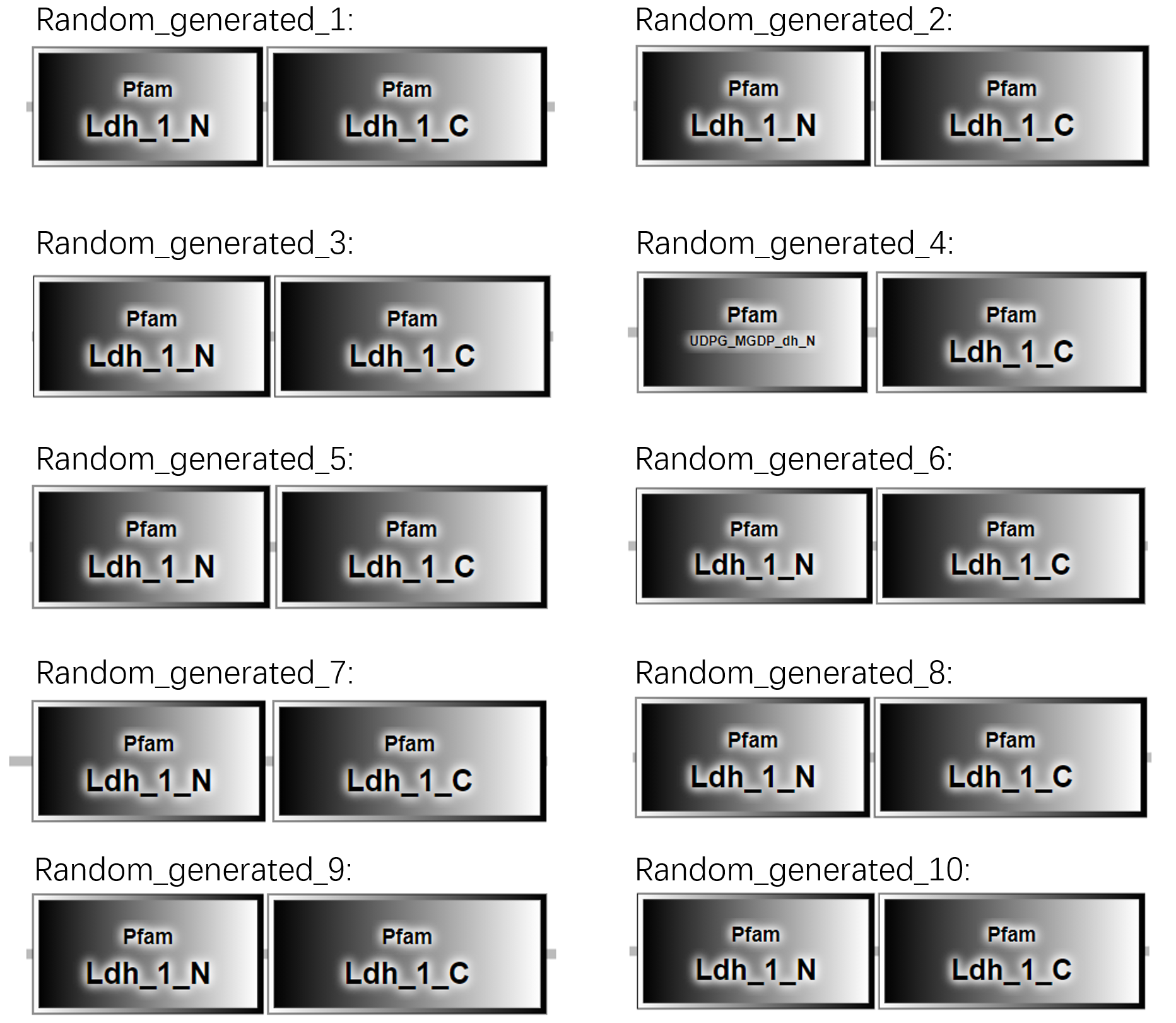
**

**Figure S4.** Pfam domains of 10 randomly generated sequences for MDH dataset. Domain ‘Ldh_1_N’ and ‘Ldh_1_C’ contain more than 100 amino acids and are distant from each other. ‘Ldh_1_N’ is the NAD binding domain and ‘Ldh_1_C’ is the α/β C-terminal domain of lactate/malate dehydrogenase. Nine out of ten generated sequences contain both domains.


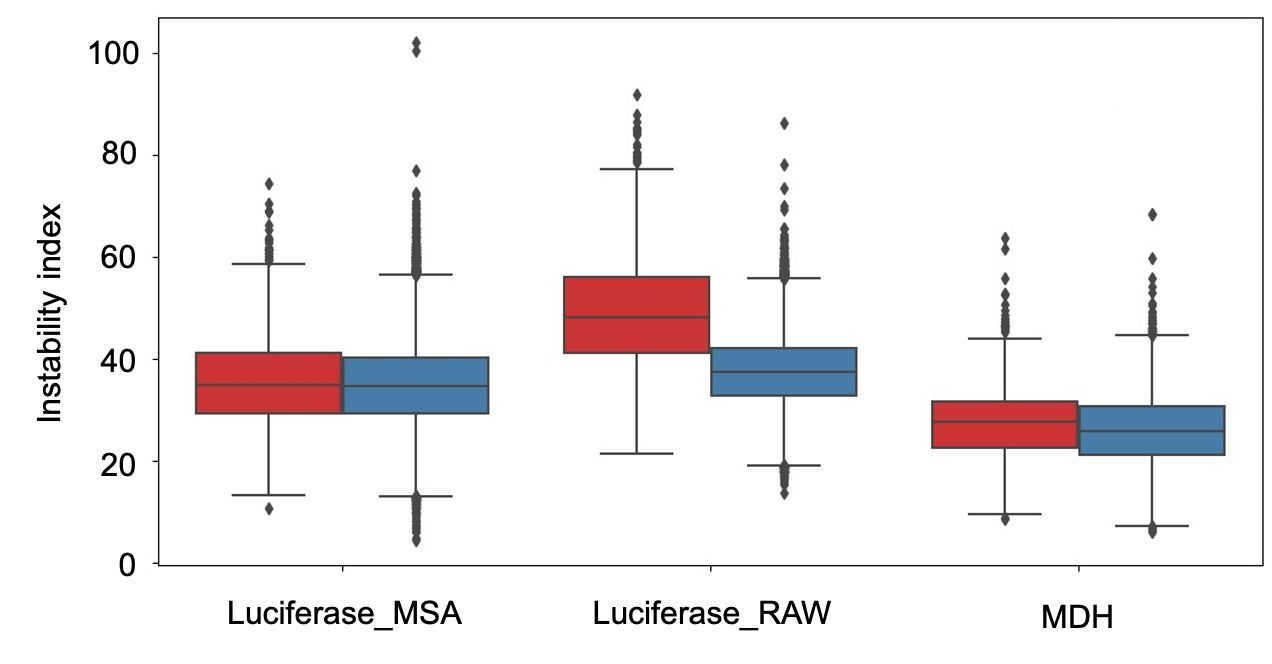


**Figure S5.** Comparison of sequence stability for natural and generated sequences in three datasets (left: Luciferase_MSA; mid: Luciferase_RAW; right: MDH; red: PRO-LDM generated sequences; blue: training sequences).

**Figure S6.** Amino acid compositions of natural and generated protein sequences. **(A)** MDH; **(B)** Luciferase_MSA; **(C)** Luciferase_RAW.


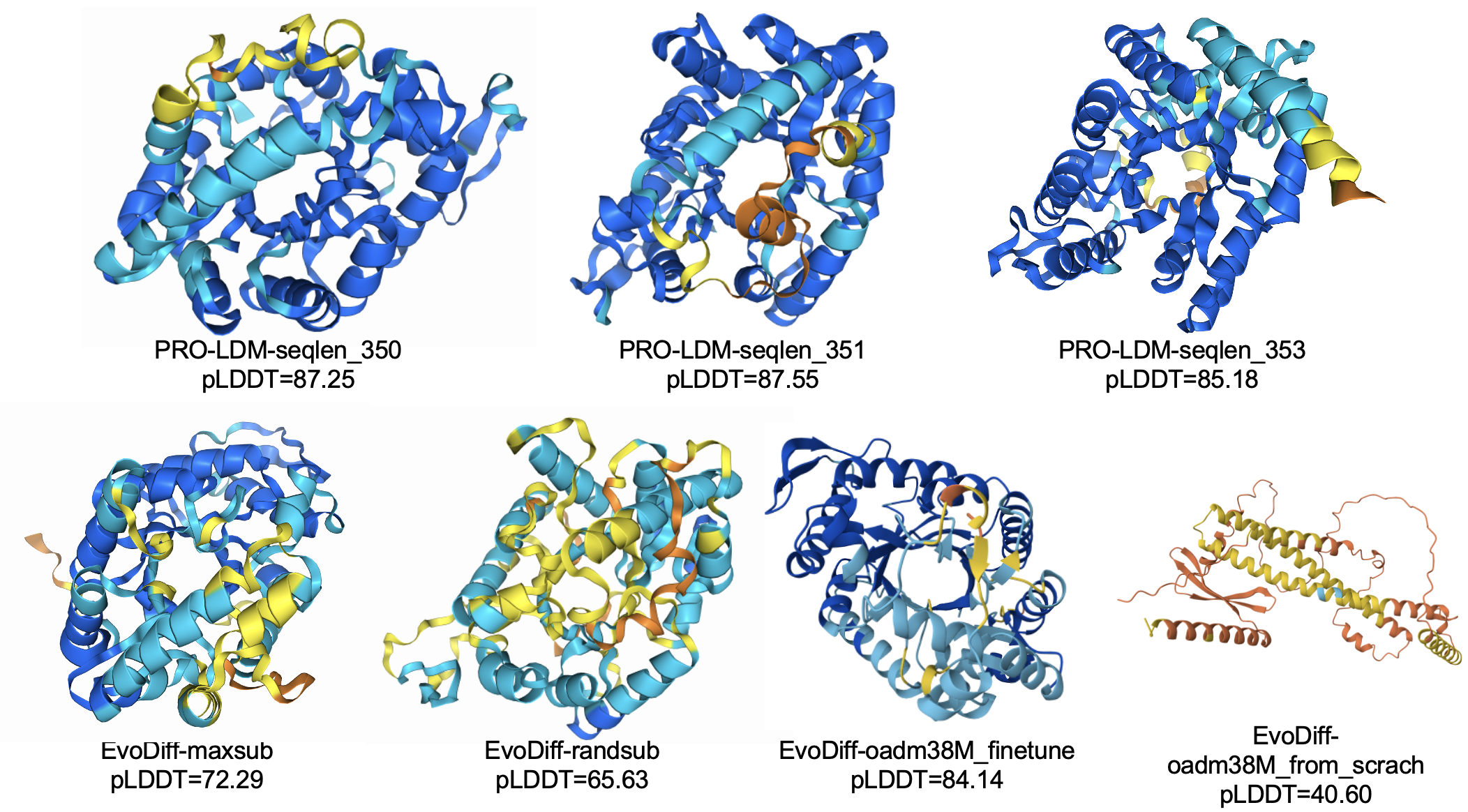


**Figure S7.** The predicted protein structures and average pLDDT scores for MSA sequences generated by PRO-LDM and EvoDiff. PRO-LDM generated MSA sequences are trained on the luciferase_MSA dataset. EvoDiff-maxsub and EvoDiff-randsub are two versions of EvoDiff. Query sequences are generated from 64 luciferase_MSA sequences using random or Max-Hamming subsampled MSAs. EvoDiff-oadm38M_finetune is fine-tuned using the pretrained weights of the oadm_38M version of EvoDiff, while EvoDiff-oadm38M_from_scratch is trained from scratch without loading any pretrained weights.


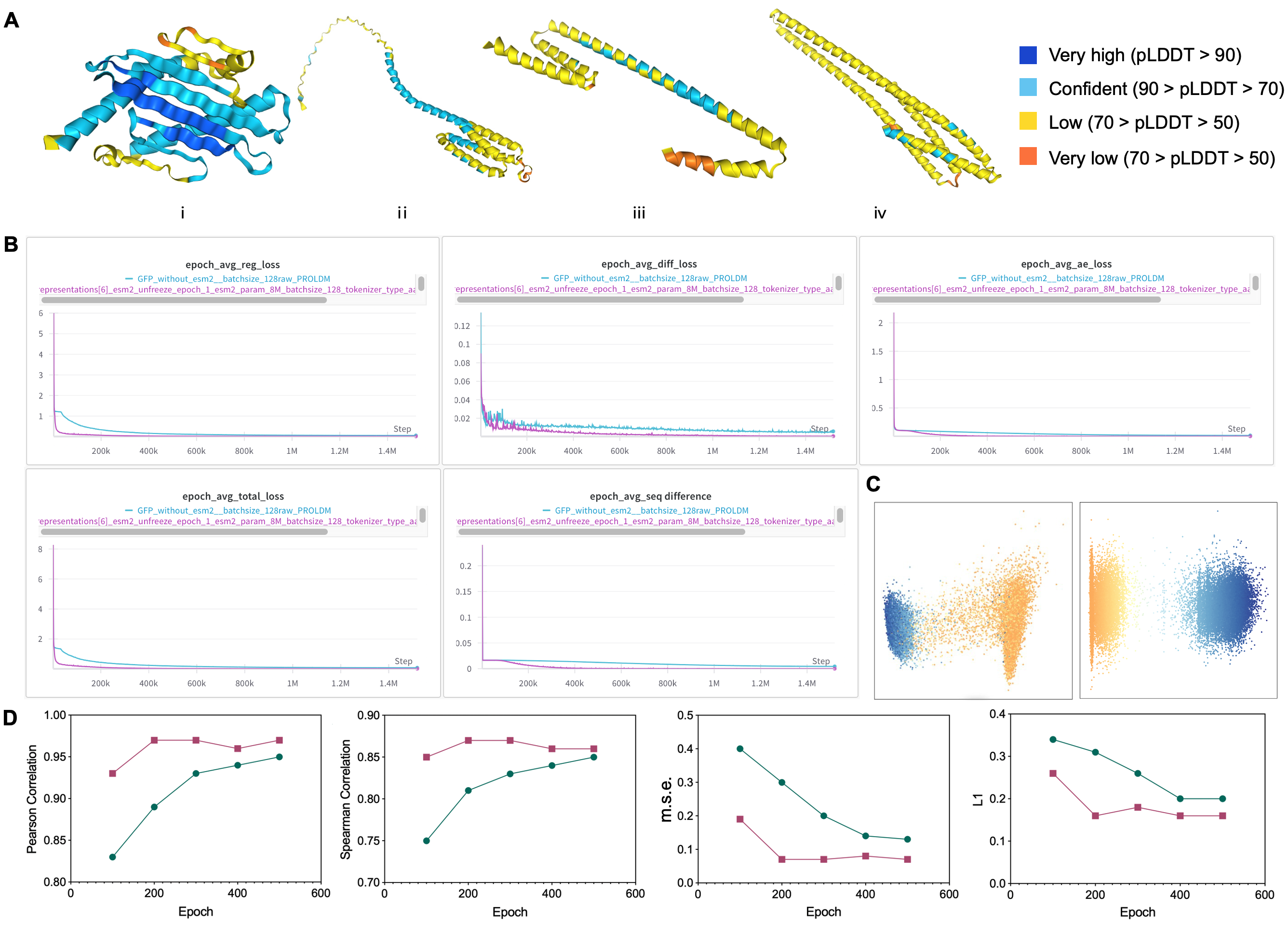


**Figure S8.** The performance comparison between PRO-LDM and PRO-LDM(ESM2). **(A)** The predicted structure of unconditional generated protein by PRO-LDM(ESM2) trained on CATH (i) or Swissprot (ii, iii, iv). Protein structure is colored to their pLDDT level. **(B)** The training loss and sequence difference during the training process. X axis: training step; Y axis: the value of loss. Purple line: PRO-LDM(ESM2); blue line: PRO-LDM. **(C)** Latent space representations of natural GFP sequences. The protein sequence representations are visualized by PCA, and each point is colored according to its corresponding fitness value. From blue to orange: high fitness to low fitness. **(D)** The fitness prediction metrics value variation during the training process. X axis: training epoch; Y axis: fitness prediction metrics value. Pink: PRO-LDM(ESM2); green: PRO-LDM.


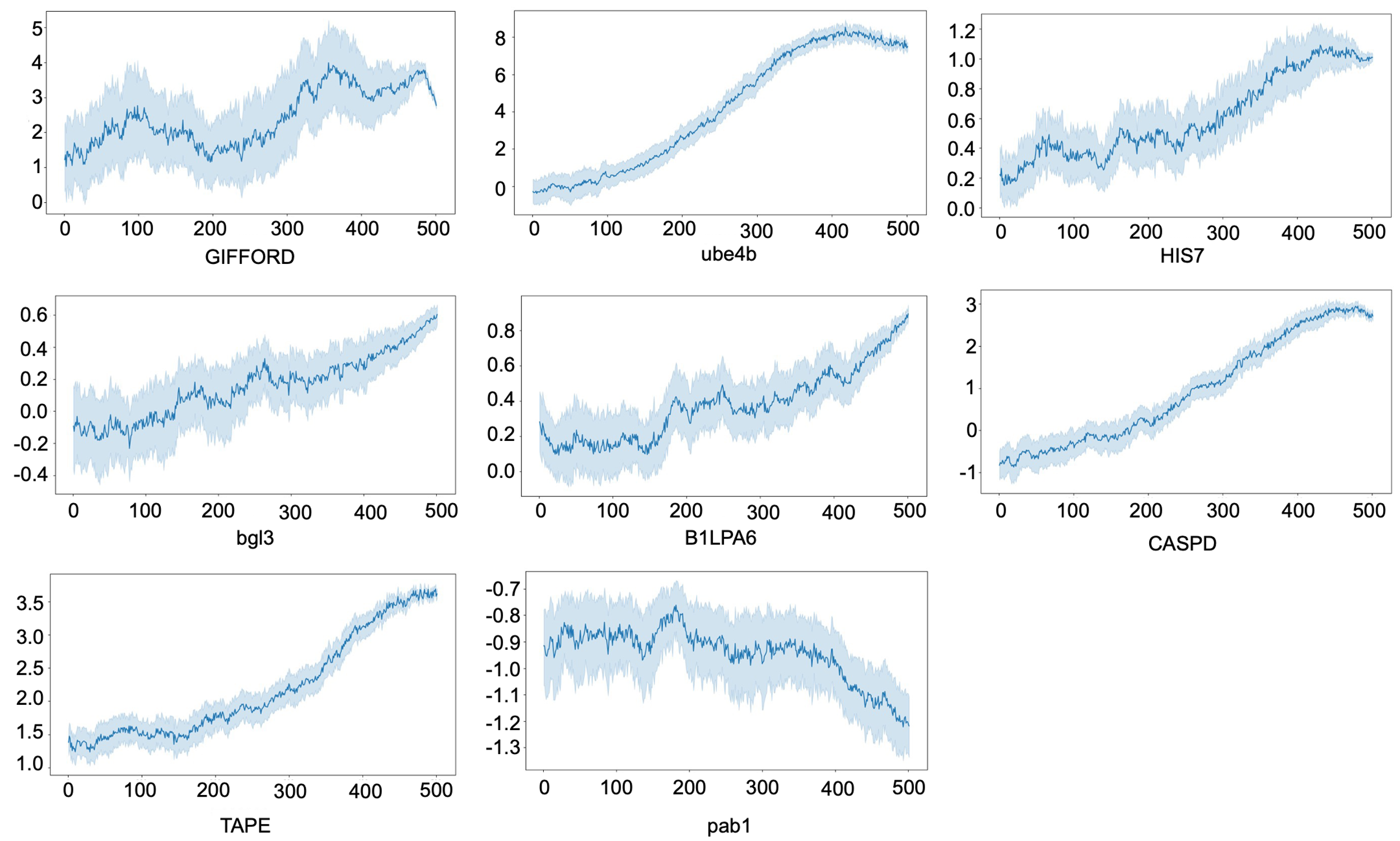


**Figure S9.** The change of predicted fitness in conditional protein sequence generation. To visualize the convergence of protein fitness into the targeted area, high-fitness protein sequences are conditionally generated in 8 labeled datasets. The protein fitness is predicted using latent variables generated during the denoising process, utilizing the pre-trained regressor. Sixty-four protein sequences are generated for each dataset. The intermediate dark blue line represents the average fitness value across 64 sequences. X axis: time step; Y axis: fitness.


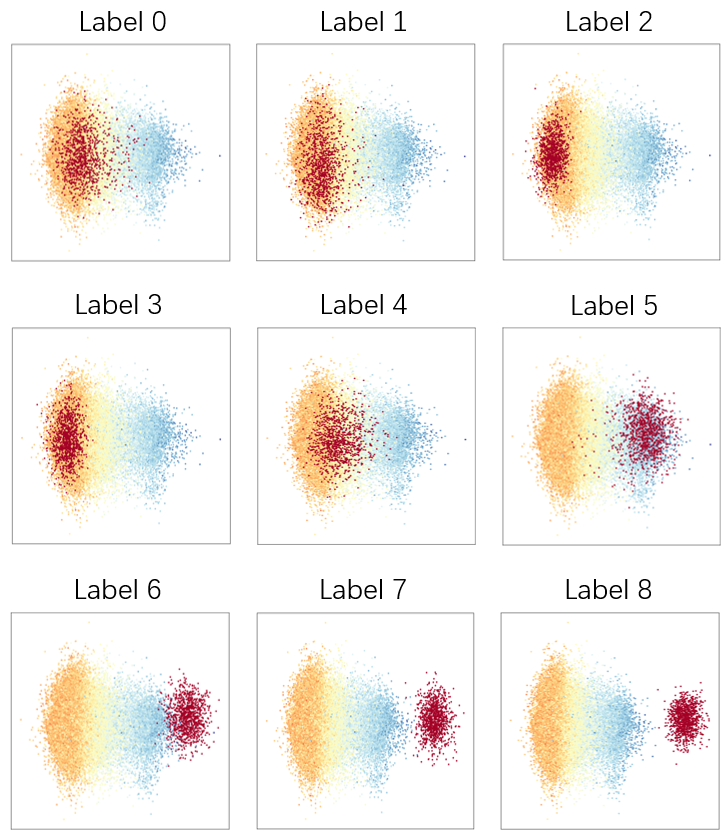


**Figure S10.** Natural sequences (cool-color: higher fitness; warm-color: lower fitness) and conditionally generated protein sequences (red) visualized in latent space (Gifford).


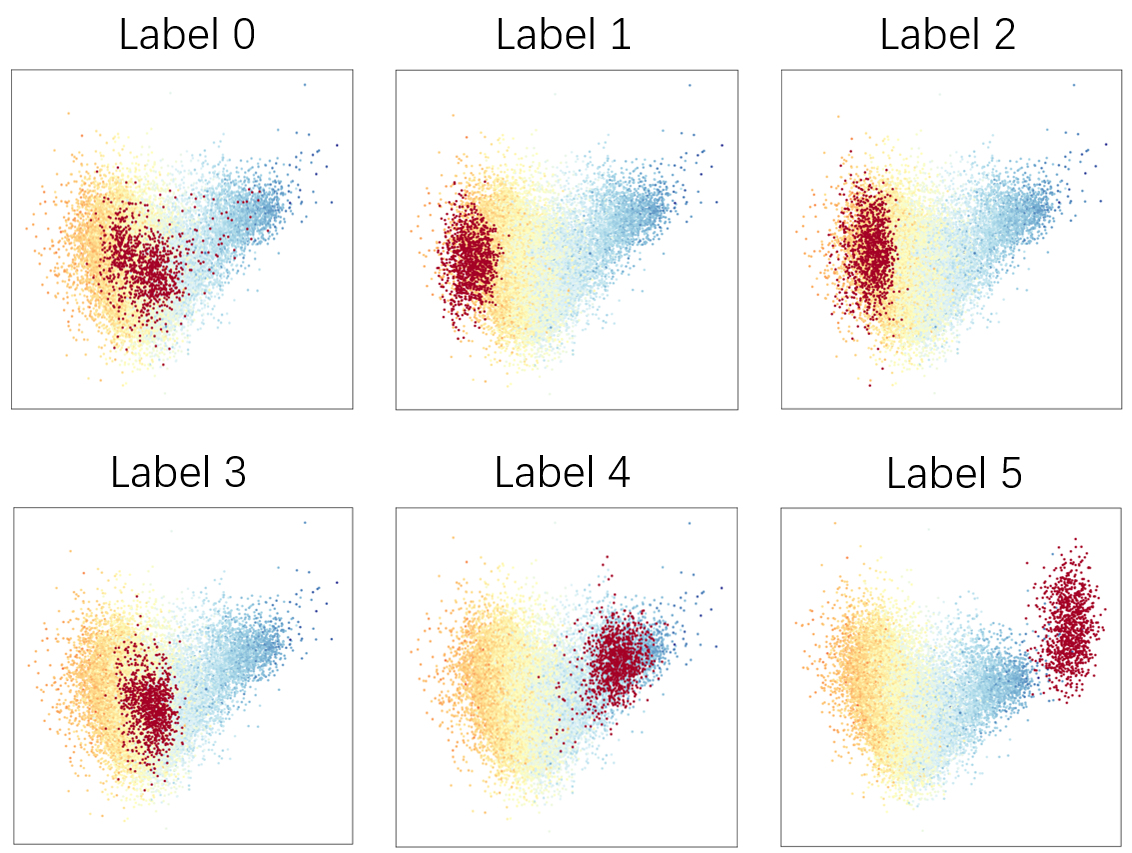


**Figure S11.** Natural sequences (cool-color: higher fitness; warm-color: lower fitness) and conditionally generated protein sequences (red) visualized in latent space (Ube4b).

**
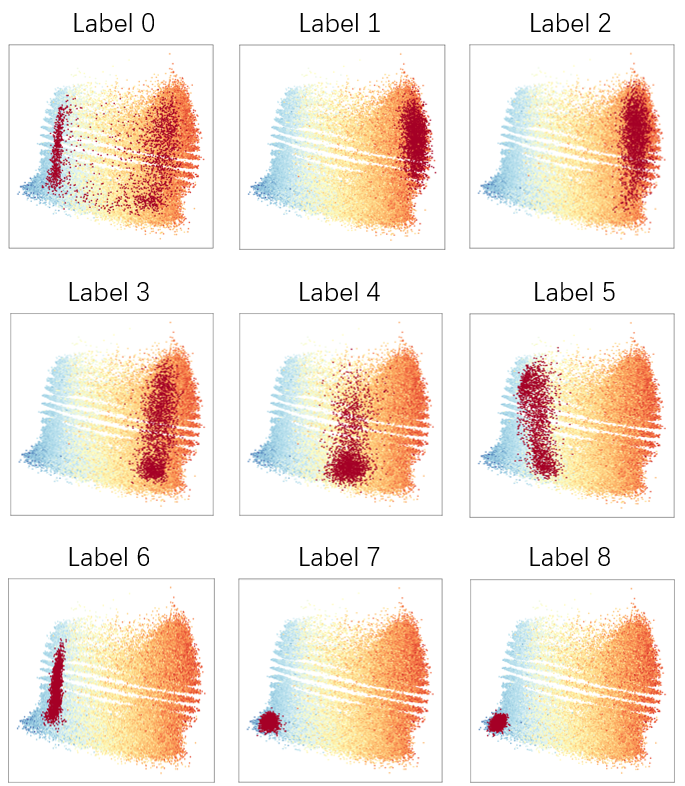
**

**Figure S12.** Natural sequences (cool-color: higher fitness; warm-color: lower fitness) and conditionally generated protein sequences (red) visualized in latent space (CAPSD).

**
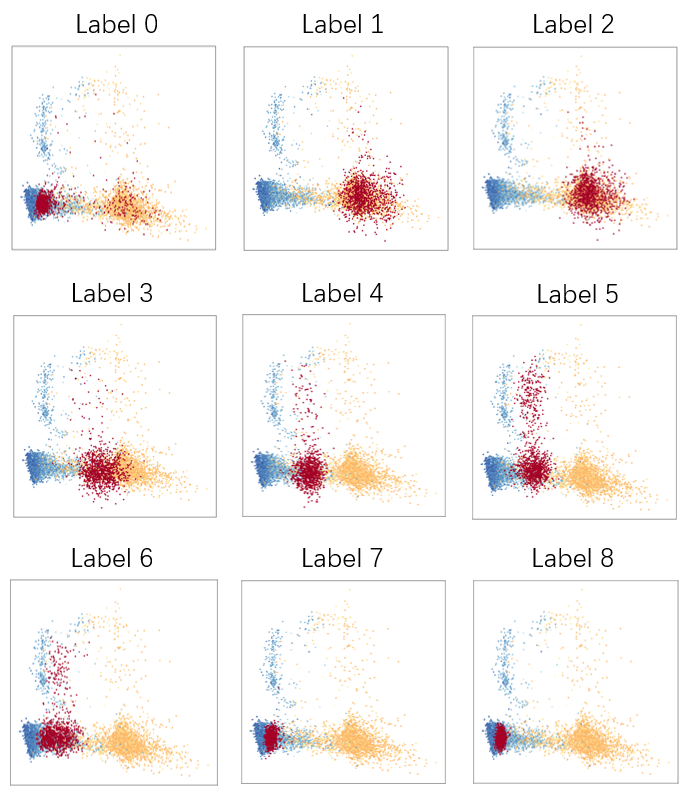
**

**Figure S13.** Natural sequences (cool-color: higher fitness; warm-color: lower fitness) and conditionally generated protein sequences (red) visualized in latent space (TAPE).

**
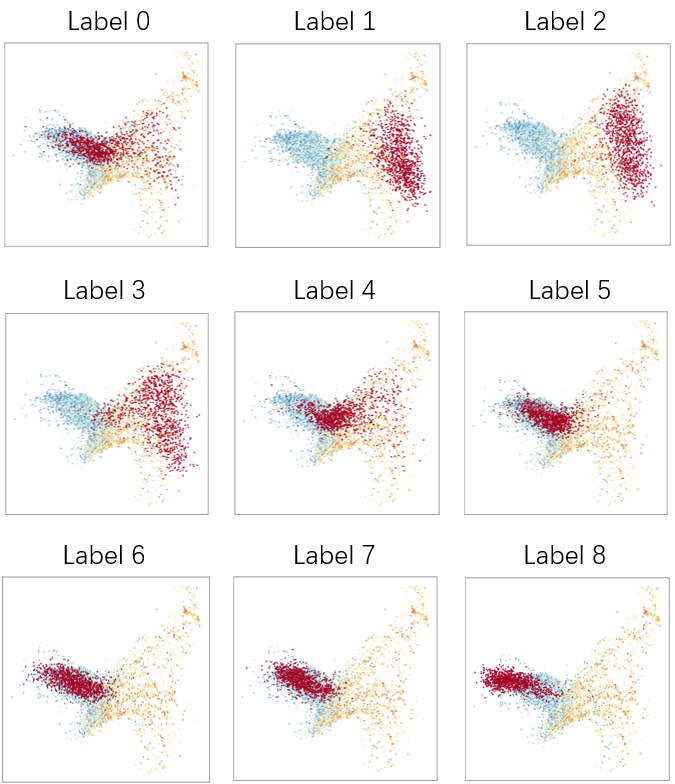
**

**Figure S14.** Natural sequences (cool-color: higher fitness; warm-color: lower fitness) and conditionally generated protein sequences (red) visualized in latent space (Bgl3).

**
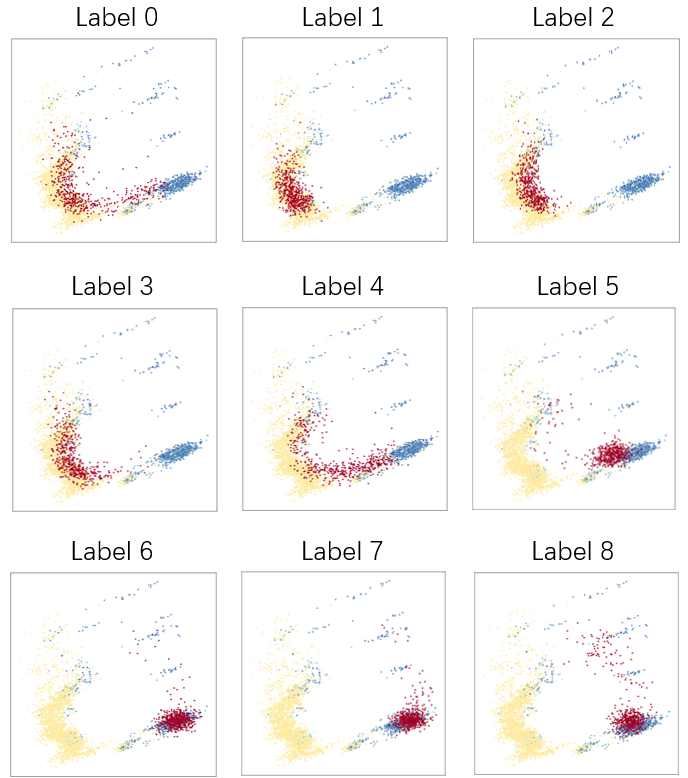
**

**Figure S15.** Natural sequences (cool-color: higher fitness; warm-color: lower fitness) and conditionally generated protein sequences (red) visualized in latent space (HIS7).

**
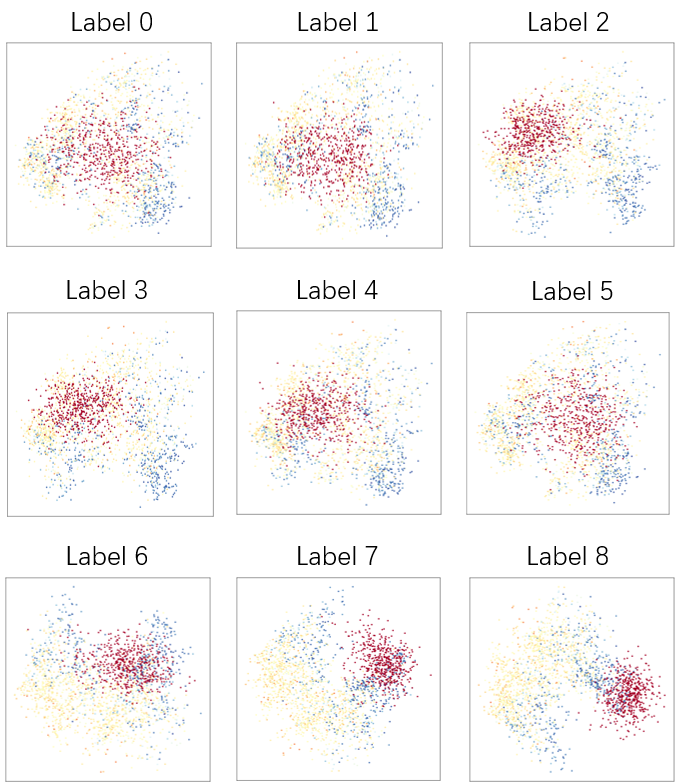
**

**Figure S16.** Natural sequences (cool-color: higher fitness; warm-color: lower fitness) and conditionally generated protein sequences (red) visualized in latent space (B1LPA6).

**
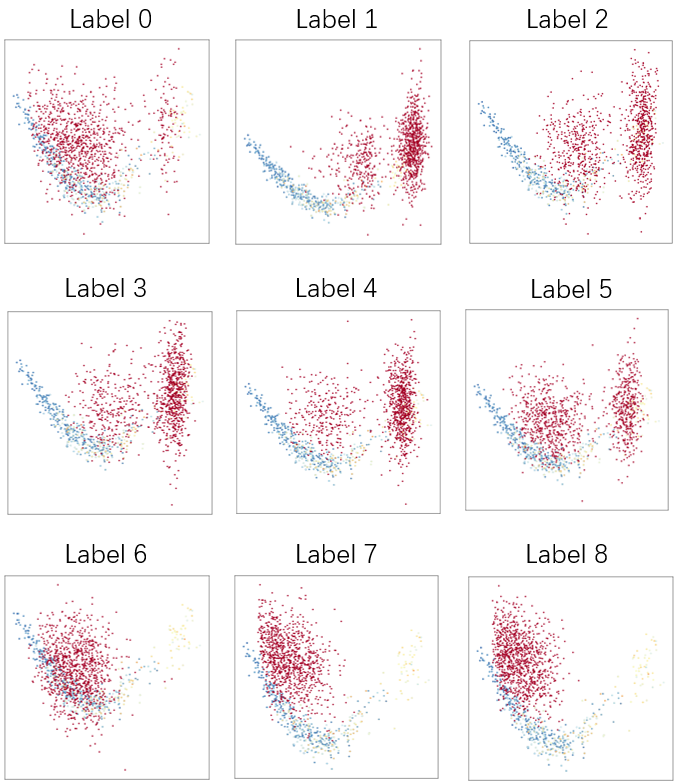
**

**Figure S17.** Natural sequences (cool-color: higher fitness; warm-color: lower fitness) and conditionally generated protein sequences (red) visualized in latent space (Pab1).

**Figure S18.** Fitness distribution of natural (blue) and generated (red) sequences of 8 mutation datasets in each label. **(A)** Gifford; **(B)** Ube4b; **(C)** CAPSD; **(D)** TAPE; **(E)** Bgl3; **(F)** HIS7; **(G)** B1LPA6; **(H)** Pab1.

**Figure S19.** Fitness distribution of generated GFP sequences by different optimization strategies of ReLSO. Y axis: predicted fitness value.

**Figure S20.** Latent space visualization of natural and generated GFP sequences embeddings by ReLSO. Light color: high fitness; dark color: low fitness; triangle: generated GFP; green circle in the subfigures: the latent space of training set.

**Figure S21.** PRO-LDM and ProteinBERT predicted fitness comparison of generated sequences. Sixty-four GFP sequences are generated from each model for comparison. X axis: fitness prediction methods; Y axis: predicted fitness value.

**Figure S22.** Visualization of the latent space from different encoders. The generated (red) and natural sequences (cool-color: higher fitness; warm-color: lower fitness) of each label are mapped into latent space and visualized by PCA. For each encoder, 1000 sequences are generated, and a limited range of values for $\omega$ and load_epoch is explored. The versions yielding the higher number of high-fitness sequences under each configuration are selected for visualization.

**Figure S23.** Outlier protein design by PRO-LDM at ω = 0.1 for GFP. **(A)** Characteristic structure for designed GFP protein predicted by AlphaFold2 and colored to pLDDT (deep blue: pLDDT > 90; light blue: 90 > pLDDT > 70; yellow: 70 > pLDDT > 50). **(B)** The number of mutation sites for 5000 generated sequences. X axis: number of mutation sites; Y axis: the frequency of occurrence. **(C)** Statistical data of the generated sequences. 5000 sequences are generated at ω = 0.1. One hundred sequences are randomly selected to calculate the average pLDDT and r.m.s.d. **(D)** Visualization of the latent space. The generated (red) and natural sequences (cool-color: high fitness; warm-color: low fitness) of each label are mapped into the latent space and visualized using PCA. **(E-G)** Histogram of predicted fitness of generated sequences against fitness values in the training set. The value used is predicted by **E**: PRO-LDM, **F**: ProteinBERT, and **G**: Tranception. The Y axis of the subplots represents the relative number of sequences with the same fitness value (purple: natural sequences; green: generated sequences); the X axis represents the fitness value.

**Figure S24.** Outlier protein design by PRO-LDM at ω = 0.5 for GFP. **(A)** Characteristic structure for designed GFP protein predicted by AlphaFold2 and colored to pLDDT (deep blue: pLDDT > 90; light blue: 90 > pLDDT > 70; yellow: 70 > pLDDT > 50). **(B)** The number of mutation sites for 5000 generated sequences. X axis: number of mutation sites; Y axis: the frequency of occurrence. **(C)** Statistical data of the generated sequences. 5000 sequences are generated at ω = 0.5. One hundred sequences are randomly selected to calculate the average pLDDT and r.m.s.d. **(D)** Visualization of the latent space. The generated (red) and natural sequences (cool-color: high fitness; warm-color: low fitness) of each label are mapped into the latent space and visualized using PCA. **(E-G)** Histogram of predicted fitness of generated sequences against fitness values in the training set. The value used is predicted by **E**: PRO-LDM, **F**: ProteinBERT, and **G**: Tranception. The Y axis of the subplots represents the relative number of sequences with the same fitness value (purple: natural sequences; green: generated sequences); the X axis represents the fitness value.


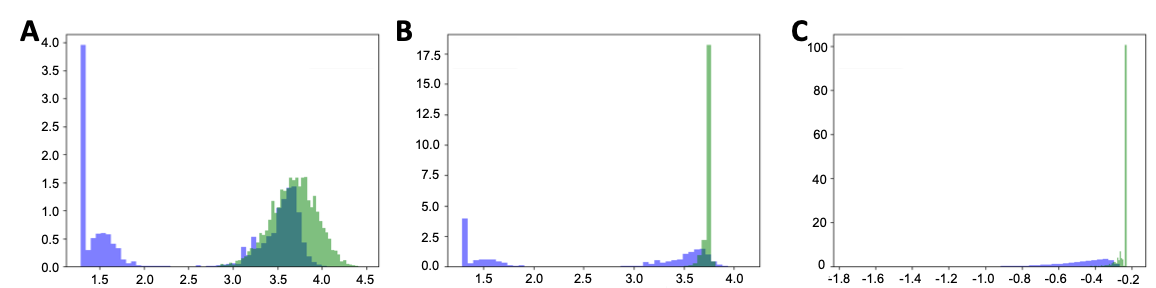


**Figure S25. (A-C)** Histogram of predicted fitness for generated sequences against fitness values in the training set at ω = 1. The value used in these three plots is predicted by A: PRO-LDM, B: ProteinBERT, and C: Tranception. The Y axis denotes relative numbers of sequences with the same fitness value (purple: natural sequences; green: generated sequences).

**Figure S26.** Outlier protein design by PRO-LDM at ω = 10 for GFP. **(A)** Characteristic structure for designed GFP protein predicted by AlphaFold2 and colored to pLDDT (deep blue: pLDDT > 90; light blue: 90 > pLDDT > 70; yellow: 70 > pLDDT > 50). **(B)** The number of mutation sites for 5000 generated sequences. X axis: number of mutation sites; Y axis: the frequency of occurrence. **(C)** Statistical data of the generated sequences. 5000 sequences are generated at ω = 10. One hundred sequences are randomly selected to calculate the average pLDDT and r.m.s.d. **(D)** Visualization of the latent space. The generated (red) and natural sequences (cool-color: high fitness; warm-color: low fitness) of each label are mapped into the latent space and visualized using PCA. **(E-G)** Histogram of predicted fitness of generated sequences against fitness values in the training set. The value used is predicted by **E**: PRO-LDM, **F**: ProteinBERT, and **G**: Tranception. The Y axis of the subplots represents the relative number of sequences with the same fitness value (purple: natural sequences; green: generated sequences); the X axis represents the fitness value.

**Figure S27.** Outlier protein design by PRO-LDM at ω = 20 for GFP. **(A)** Characteristic structure for designed GFP protein predicted by AlphaFold2 and colored to pLDDT (deep blue: pLDDT > 90; light blue: 90 > pLDDT > 70; yellow: 70 > pLDDT > 50). **(B)** The number of mutation sites for 5000 generated sequences. X axis: number of mutation sites; Y axis: the frequency of occurrence. **(C)** Statistical data of the generated sequences. 5000 sequences are generated at ω = 20. One hundred sequences are randomly selected to calculate the average pLDDT and r.m.s.d. **(D)** Visualization of the latent space. The generated (red) and natural sequences (cool-color: high fitness; warm-color: low fitness) of each label are mapped into the latent space and visualized using PCA. **(E-G)** Histogram of predicted fitness of generated sequences against fitness values in the training set. The value used is predicted by **E**: PRO-LDM, **F**: ProteinBERT, and **G**: Tranception. The Y axis of the subplots represents the relative number of sequences with the same fitness value (purple: natural sequences; green: generated sequences); the X axis represents the fitness value.

**Figure S28.** Distribution of mutation across GFP sequence generated with varying ω values. X axis: ω values; Y axis: mutation counts.

**Figure S29.** Structure predictions by AlphaFold3 of selected PRO-LDM generated sequences at ω = 20-100 for GFP.

**Figure S30.** Outlier protein design by PRO-LDM at ω = 40 for GFP. **(A)** Characteristic structure for designed GFP protein predicted by AlphaFold2 and colored to pLDDT (deep blue: pLDDT > 90; light blue: 90 > pLDDT > 70; yellow: 70 > pLDDT > 50). **(B)** The number of mutation sites for 5000 generated sequences. X axis: number of mutation sites; Y axis: the frequency of occurrence. **(C)** Statistical data of the generated sequences. 5000 sequences are generated at ω = 40. One hundred sequences are randomly selected to calculate the average pLDDT and r.m.s.d. **(D)** Visualization of the latent space. The generated (red) and natural sequences (cool-color: high fitness; warm-color: low fitness) of each label are mapped into the latent space and visualized using PCA. **(E-G)** Histogram of predicted fitness of generated sequences against fitness values in the training set. The value used is predicted by **E**: PRO-LDM, **F**: ProteinBERT, and **G**: Tranception. The Y axis of the subplots represents the relative number of sequences with the same fitness value (purple: natural sequences; green: generated sequences); the X axis represents the fitness value.


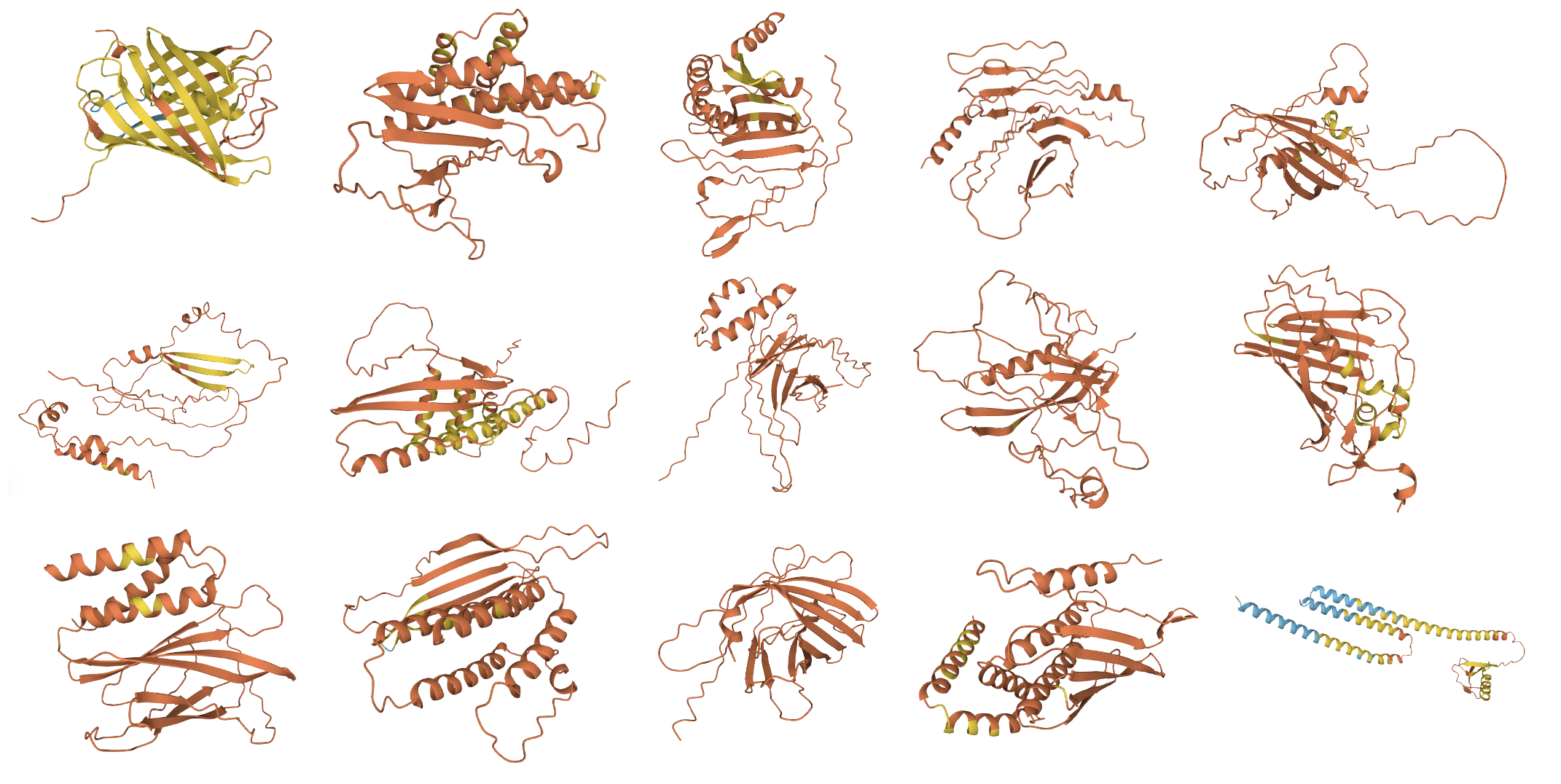


**Figure S31.** Structure predictions by AlphaFold2 of selected PRO-LDM generated sequences at ω = 1000 for GFP.


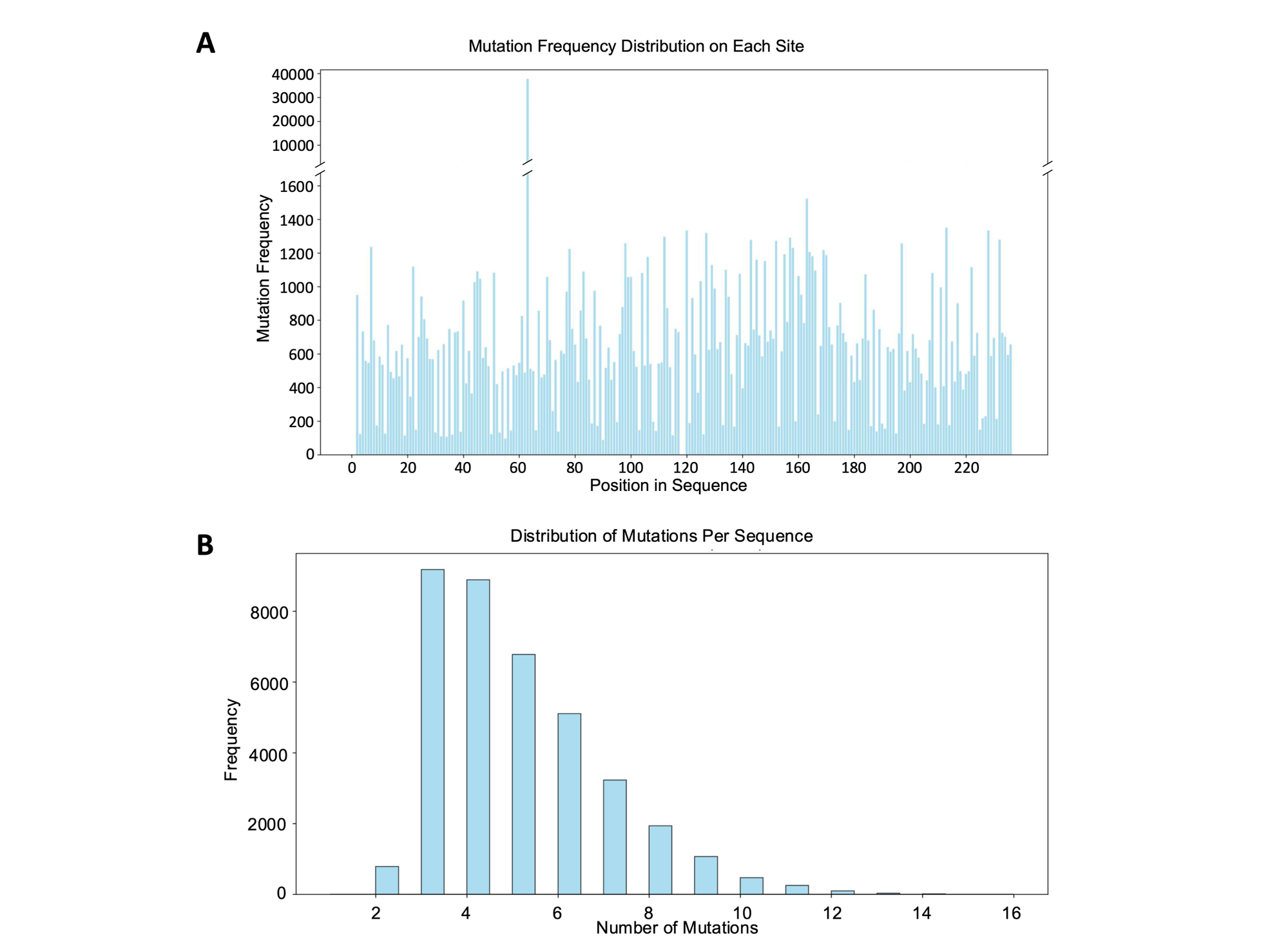


**Figure S32.** Analysis of mutation sites and mutation counts in the training set (in a total of 37816 mutants). (**A**) Mutation frequency distribution on each site. (**B**) Distribution of mutations per sequence.


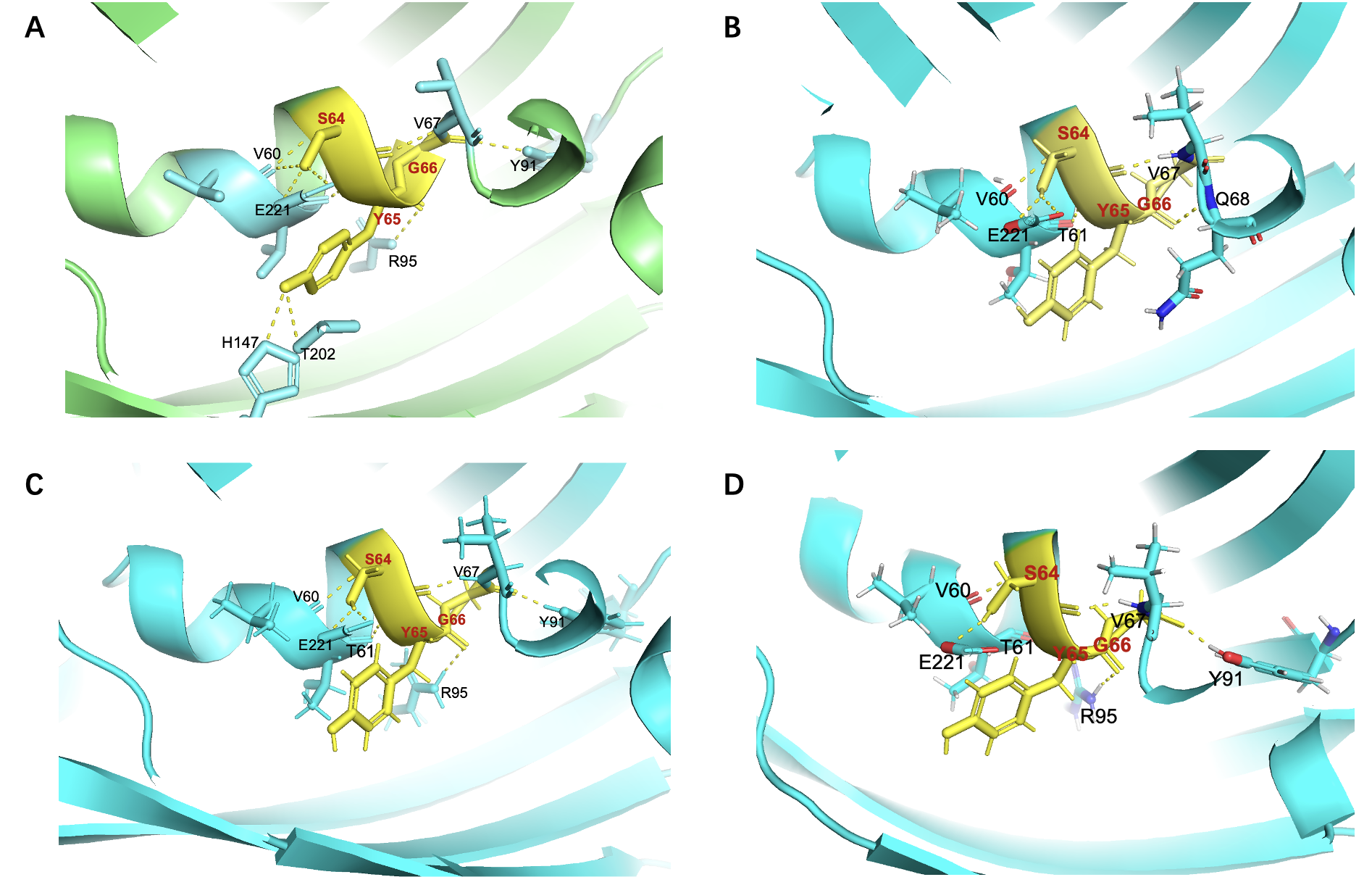


**Figure S33.** Visualization of hydrogen bond at the chromophore center. **(A)** wt-GFP; **(B)** pro_H; **(C)** pro_1498; **(D)** pro_2421.

**Figure S34.** Structural predictions of pro_1498 and pro_2421 with annotated polar interactions at mutation sites. Orange: chromophore; blue: mutation sites.

**Figure S35.** Gel-electrophoresis image of four GFP variants in soluble and pellet fractions. SF: soluble fraction, PF: pellet fraction.

**Supplement Tables**

**Table S1. The comparison for computing power requirement between PRO-LDM and mainstream protein sequence design models.**

| **Model** | **Dataset** | **Training** | **Sampling** | **Model Type** | **Model Information** | **Ref.** |
| --- | --- | --- | --- | --- | --- | --- |
| ProtGPT2 | Uniref50 | 128 × A100(4 days) | - | Transformer decoder | 738 M parameters | ^1^ |
| MSA Transformer | Uniref50 | 1 × V100 | - | Transformer encoder | 100M parameters | ^2^ |
| UniRep | Uniref50 | 4 × K80 (4 weeks) | - | mLSTM | 18.2 M parameters | ^3^ |
| ProteinGAN | MDH | 1 × P100(9 days) | - | GAN | 60 M parameters | ^4^ |
| ESM-2 | UniRef50 | over 512 × V100 GPUs (8/30/60 days) | - | Transformer encoder | 150M/3B/15B parameters | ^5^ |
| EvoDiff | UniRef50 | 8 × V100 (~2 weeks)  32 × V100 (12-23+ days) | 2.4-5.5 min for 64 seqs | Diffusion model | 38M parameters  640M parameters | ^6^ |
| DPLM-2 | PDB and Swissprot (200,000 proteins) | 8 × A100 (3 days for 150M), 16 A100 (a week for 650M) | - | Diffusion model | 150M and 650M parameters | ^7^ |
| DiMA | Swissprot or AFDBv4-90 | 4 × A100 (10 days) | - | Diffusion model | 33M | ^8^ |
| ReLSO | Various datasets | 8 × A800 (6 hours and 21min) (our reproduction)  2 ×TITAN RTX graphics (original report) | 3 hours and 50 min for 6×30 seqs | Transformer encoder | 1.4M parameters | ^9^ |
| PRO-LDM | Various datasets | 4 × V100(5 hours) | 3 min for 64 seqs | Diffusion model, Transformer encoder | 22M parameters | This work |
| PRO-LDM-ESM2 | Swissprot (572,214 proteins) | 1 × 4090 (50 hours) | 3 min for 64 seqs |  | 32M parameters | This work |

**Table S2. Mann-Whitney U test of amino acids with similar and different biochemical properties.** Based on the amino acid types in the second column, a Mann-Whitney U test was performed to determine the statistical significance of the observed differences between each type of amino acids against all other types. The amino acid embeddings were from same proteins were used as in Figure S1.

|  | **Amino acid type** | **P-value** |
| --- | --- | --- |
| Luciferase_MSA | hydrophobic aliphatic | 1.68e-08 |
|  | hydrophobic aromatic | 2.68e-07 |
|  | polar neutral | 1.13e-06 |
|  | charge basic | 2.45e-05 |
|  | charge acidic | 8e-04 |
| Luciferase_RAW | hydrophobic aliphatic | 2.58e-06 |
|  | hydrophobic aromatic | 0.15 |
|  | polar neutral | 1.66e-05 |
|  | charge basic | 6.25e-05 |
|  | charge acidic | 8e-04 |
| MDH | hydrophobic aliphatic | 1.04e-08 |
|  | hydrophobic aromatic | 8.52e-07 |
|  | polar neutral | 2.67e-06 |
|  | charge basic | 5.20e-05 |
|  | charge acidic | 8e-04 |

**Table S3. Deep mutational scanning (DMS) datasets used in conditional PRO-LDM.**

|  | **Datasets** | **Description** | **Organism** | **Molecular function** | **length** | **Variants** | **Ref** |
| --- | --- | --- | --- | --- | --- | --- | --- |
| Substitutions | Gifford | CDR3 of human Immunoglobulin G antibodies | human | IgG binding | 20 | 90,459 | ^10^ |
|  | GFP | Green fluorescent  protein | *Aequorea victoria* | Fluorescence | 237 | 54,025 | ^11^ |
|  | TAPE | Green fluorescent  protein | *Aequorea victoria* | Fluorescence | 237 | 54,025 | ^12^ |
|  | Bgl3 | β-glucosidase | *Streptococcus* sp. | Hydrolysis of β-glucosidic linkages | 501 | 26,653 | ^13^ |
|  | Pab1 | RNA recognition motif (RRM) domain | *Saccharomyces cerevisiae* | Poly(A) binding | 75 | 40,852 | ^14^ |
|  | Ube4b | Ubiquitination factor E4B U-box domain | *Mus musculus* | Ubiquitin-activating enzyme activity | 102 | 98,297 | ^15^ |
| Indels | HIS7 | Imidazoleglycerol-phosphate dehydratase | yeast | catalyzing the sixth step in the histidine biosynthesis pathway | 206-235 | 6,102 | ^16^ |
|  | CAPSD | Capsid protein | Adeno-associated virus | delivery vectors for gene therapy | 735-749 | 250,907 | ^17^ |
|  | B1LPA6 | Bifunctional chorismate mutase/prephenate dehydratase | - | Catalyzes the Claisen rearrangement and the decarboxylation/dehydration | 80-95 | 3,074 | ^18^ |

**Table S4. Fitness prediction metrics for DMS datasets.**

| **Model** | **Dataset** | **Pearson’r** | **Spearman** | **m.s.e** | **L1** |
| --- | --- | --- | --- | --- | --- |
| PRO-LDM | Bgl3 | 0.49 | 0.44 | 1.52 | 0.97 |
| JT-AE |  | 0.51 | 0.47 | 1.86 | 1.07 |
| PRO-LDM | GFP | 0.95 | 0.84 | 0.12 | 0.19 |
| JT-AE |  | 0.90 | 0.81 | 0.33 | 0.37 |
| PRO-LDM | Pab1 | 0.43 | 0.39 | 3.19 | 1.51 |
| JT-AE |  | 0.43 | 0.39 | 3.06 | 1.44 |
| PRO-LDM | Gifford | 0.82 | 0.47 | 0.26 | 0.38 |
| JT-AE |  | 0.83 | 0.47 | 0.23 | 0.34 |
| PRO-LDM | TAPE | 0.87 | 0.65 | 0.95 | 0.88 |
| JT-AE |  | 0.63 | 0.56 | 1.27 | 0.79 |
| PRO-LDM | Ube4b | 0.65 | 0.61 | 2.24 | 1.15 |
| JT-AE |  | 0.60 | 0.56 | 2.89 | 1.30 |
| PRO-LDM | HIS7 | 0.83 | 0.69 | 0.07 | 0.17 |
| JT-AE |  | 0.90 | 0.75 | 0.04 | 0.11 |
| PRO-LDM | CAPSD | 0.95 | 0.90 | 1.26 | 0.79 |
| JT-AE |  | 0.94 | 0.88 | 1.33 | 0.80 |
| PRO-LDM | B1LPA6 | 0.58 | 0.51 | 0.12 | 0.27 |
| JT-AE |  | 0.61 | 0.54 | 0.12 | 0.26 |

**Table S5. Correlation of Shannon entropy between natural and generated sequences.**

| **Model** | **Dataset** | **Pearson’r** | | **P-value** | **m.s.e** | |
| --- | --- | --- | --- | --- | --- | --- |
| PRO-LDM | MDH | | 0.9745 | 3.1653e-53 | 0.0253 | |
| VAE |  |  | 0.6689 | 1.1514e-64 | 0.2840 | |
| JT-AE |  | | 0.0470 | 2.4627e-4 | 0.3473 |  |
| PRO-LDM | Luciferase_MSA | | 0.9921 | 8.6748e-20 | 0.0079 | |
| VAE |  |  | 0.9768 | 1.8404e-59 | 0.0226 | |
| JT-AE |  | | 0.2042 | 7.7419e-22 | 0.4064 |  |
| PRO-LDM | Luciferase_RAW | | 0.7634 | 0.63 | 0.0851 | |
| VAE |  |  | 0.8832 | 1.1809e-53 | 0.0910 | |
| JT-AE |  | | 0.3042 | 7.5856e-50 | 0.4191 |  |

**Table S6. Average Pearson’s correlation coefficient of amino-acid pair frequencies between natural and generated sequences.**

| **Dataset** | **PRO-LDM** | **VAE** |
| --- | --- | --- |
| MDH | 0.9852 | 0.8504 |
| Luciferase_MSA | 0.8823 | 0.8296 |
| Luciferase_RAW | 0.8525 | 0.7970 |

**Table S7.** **The performance comparison of PRO-LDM, ProteinMPNN and EvoDiff.**

| **Model** | **ReconKL** | **Minimum Hamming Distance** | **r.m.s.d. against pro_H** | **Seq avg. pLDDT** |
| --- | --- | --- | --- | --- |
| EvoDiff-oadm-38M (from scratch) | 1.45e-2 | 0.31±0.032 | 0.506±0.069 | 74.188±3.977 |
| EvoDiff-oadm-38M (finetune) | 1.76e-4 | 0.012±0.008 | **0.159±0.008** | 91.192±1.685 |
| EvoDiff-D3PM_BLOSUM-38M (from scratch) | 3.71e-4 | 0.007±0.005 | 0.167±0.011 | 91.852±1.488 |
| EvoDiff-D3PM_BLOSUM-38M (finetune) | 2.65e-4 | 0.012±0.020 | 0.166±0.006 | **92.7±0.205** |
| EvoDiff-D3PM_uniform-38M (from scratch) | 1.37e-3 | 0.030±0.011 | 0.169±0.010 | 92.694±0.865 |
| EvoDiff-D3PM_uniform-38M (finetune) | 3.24e-4 | 0.011±0.007 | 0.159±0.013 | 92.658±0.431 |
| ProteinMPNN | 2.62e-2 | 0.331±0.022 | 0.292±0.028 | 92.226±0.996 |
| ESM3-medium-2024-8 | 7.64e-2 | **0.451±0.027** | 0.265±0.043 | 92.321±0.578 |
| ProGen2-small | **1.26e-5** | 0.012±0.007 | 0.162±0.006 | 91.977±0.794 |
| PRO-LDM-tiny-epoch1000-w1.8 | 2.87e-4  **1.05e-4^*^** | 0.004±0.002 | 0.188±0.010 | 90.408±0.214 |

* ReconKL for PRO-LDM was calculated in correspondence to label 8.

**Table S8. Cluster numbers of 5000 generated sequences with different hyperparameter ω created by MMseq2.** For each ω value, 5000 sequences were generated and analyzed by MMseq2 using various similarity thresholds within clusters listed. The table presented the quantity of clusters that indicated the diversity of the produced sequences.

| **Sequence identity\threshold** | **0** | **0.3** | **0.6** | **0.9** | **1** |
| --- | --- | --- | --- | --- | --- |
| $=0.1$ | 1 | 1 | 1 | 1 | 776 |
| $=0.2$ | 1 | 1 | 1 | 1 | 753 |
| $=0.3$ | 1 | 1 | 1 | 1 | 729 |
| $=0.4$ | 1 | 1 | 1 | 1 | 751 |
| $=0.5$ | 1 | 1 | 1 | 1 | 664 |
| $=0.6$ | 1 | 1 | 1 | 1 | 697 |
| $=0.7$ | 1 | 1 | 1 | 1 | 664 |
| $=0.8$ | 1 | 1 | 1 | 1 | 645 |
| $=0.9$ | 1 | 1 | 1 | 1 | 615 |
| $=1$ | 1 | 1 | 1 | 1 | 604 |
| $=2$ | 1 | 1 | 1 | 1 | 691 |
| $=3$ | 1 | 1 | 1 | 1 | 895 |
| $=4$ | 1 | 1 | 1 | 1 | 1330 |
| $=5$ | 1 | 1 | 1 | 1 | 1974 |
| $=10$ | 1 | 1 | 1 | 2 | 4694 |
| $=20$ | 1 | 1 | 1 | 1175 | 5000 |
| $=40$ | 1 | 1 | 10 | 4559 | 5000 |

**Table S9. The counts of generated outlier sequences with r.m.s.d. value at each range against pro_H**

|  | <1Å | 1-2Å | >2Å |
| --- | --- | --- | --- |
| Statistics | 137 | 31 | 12 |

**Table S10. Mutations in training sequences most similar to designed GFP variants**

|  | **Mutation sites in the closest training set counterparts** |
| --- | --- |
| **pro_1498** | R106K, A107T, R125K |
|  | A107T, V122I, R125K |
|  | R106E, V122I, R125K |
|  | A107T, R112K, R125K |
| **pro_2421** | F144Y, A162V, R183Q, E213K |
|  | F144Y, A162V, R183Q, V205A |
|  | Y73H, F144Y, A162V, R183Q |
|  | A162V, R183Q, V205A, E213K |

**Table S11. Protein sequences for wt-GFP, pro_H (highest fitness in DMS dataset), and two generated sequences** at ω = 20. Mutation sites against wt-GFP were made bold.

| Protein name | Protein sequence |
| --- | --- |
| wt-GFP (P42212) | SKGEELFTGVVPILVELDGDVNGHKFSVSGEGEGDATYGKLTLKFICTTGKLPVPWPTLVTTFSYGVQCFSRYPDHMKQHDFFKSAMPEGYVQERTIFFKDDGNYKTRAEVKFEGDTLVNRIELKGIDFKEDGNILGHKLEYNYNSHNVYIMADKQKNGIKVNFKIRHNIEDGSVQLADHYQQNTPIGDGPVLLPDNHYLSTQSALSKDPNEKRDHMVLLEFVTAAGITHGMDELYK |
| pro_H | SKGEELFTGVVPILVELDGDVNGHKFSVSGEGEGDA**S**YG**R**LTLKFICTTGKLPVPWPTLVTT**L**SYGVQCFSRYPDHMKQHDFFKSAMPEGYVQERTIFFKDDG**S**YKTRAEVKFEGDTLVNRIELKGIDFKEDGNILGHKLEYNYNSHNVYIMADKQKNGIKVNFKIRHNIEDGSVQLADHYQQNTPIGDGPVLLPDNHYLSTQSALSKDPNEKRDHMVLLEFVTAAGITHGMDELYK |
| pro_1498 | SKGEELFTGVVPILVELDGDVNGHKFSVSGEGEGDATYGKLTLKFICTTGKLPVPWPTLVTT**L**SYGVQCFSRYPDHMKQHDFFKSAMPEGYVQERTIFFKDDGNY**RA**RAEV**R**FEGDTLVNR**V**EL**R**GIDFKEDGNILGHKLEYNYNSHNVYIMADKQKNGIKVNFKIRHNIEDGSVQLADHYQQNTPIGDGPVLLPDNHYLSTQSALSKDPNEKRDHMVLLEFVTAAGITHGMDELYK |
| pro_2421 | SKGEELFTGVVPILVELDGDVNGHKFSVSGEGEGDATYGKLTLKFICTTGKLPVPWPTLVTT**L**SYGVQCFSRYPDHMKQHDFFKSAMPEGYVQERTIFFKDDGNYKTRAEVKFEGDTLVNRIELKGIDFKEDGNILGHKLEYN**F**NSHNVYIMADKQKNGIK**A**NFKIRHNIEDGSVQLADHYQ**R**NTPIGDGPVLLPDNHYLSTQS**V**LSKDPNE**E**RDHMVLLEFVTAAGITHGMDELYK |

**Table S12. Four GFP variants’ physicochemical properties.**

|  | **wt_GFP** | **pro-H** | **pro-1498** | **pro-2421** |
| --- | --- | --- | --- | --- |
| **Quantum Yield (absolute)** | 0.685 | 0.939 | 0.823 | 0.932 |
| **Extinction Coefficient** | 29035 | 29150 | 28401 | 35437 |
| **pKa** | 5.40 | 5.11 | 5.02 | 5.08 |

**SI References**

1 Ferruz, N., Schmidt, S. & Höcker, B. ProtGPT2 is a deep unsupervised language model for protein design. *Nature communications* **13**, 4348 (2022).

2 Rao, R. M. *et al.* in *Proceedings of the 38th International Conference on Machine Learning* Vol. 139 (eds Meila Marina & Zhang Tong) 8844--8856 (PMLR, Proceedings of Machine Learning Research, 2021).

3 Alley, E. C., Khimulya, G., Biswas, S., AlQuraishi, M. & Church, G. M. Unified rational protein engineering with sequence-based deep representation learning. *Nature Methods* **16**, 1315-1322 (2019). https://doi.org:10.1038/s41592-019-0598-1

4 Repecka, D. *et al.* Expanding functional protein sequence spaces using generative adversarial networks. *Nature Machine Intelligence* **3**, 324-333 (2021). https://doi.org:10.1038/s42256-021-00310-5

5 Lin, Z. *et al.* Evolutionary-scale prediction of atomic-level protein structure with a language model. *Science* **379**, 1123-1130 (2023). https://doi.org:doi:10.1126/science.ade2574

6 Alamdari, S. *et al.* Protein generation with evolutionary diffusion: sequence is all you need. *bioRxiv*, 2023.2009. 2011.556673 (2023).

7 Wang, X. *et al.* Dplm-2: A multimodal diffusion protein language model. *arXiv preprint arXiv:2410.13782* (2024).

8 Meshchaninov, V. *et al.* Diffusion on language model embeddings for protein sequence generation. *arXiv preprint arXiv:2403.03726* (2024).

9 Castro, E. *et al.* Transformer-based protein generation with regularized latent space optimization. *Nature Machine Intelligence* **4**, 840-851 (2022).

10 Liu, G. *et al.* Antibody complementarity determining region design using high-capacity machine learning. *Bioinformatics* **36**, 2126-2133 (2020).

11 Sarkisyan, K. S. *et al.* Local fitness landscape of the green fluorescent protein. *Nature* **533**, 397-401 (2016).

12 Rao, R., Meier, J., Sercu, T., Ovchinnikov, S. & Rives, A. Transformer protein language models are unsupervised structure learners. *Biorxiv*, 2020.2012. 2015.422761 (2020).

13 Romero, P. A., Tran, T. M. & Abate, A. R. Dissecting enzyme function with microfluidic-based deep mutational scanning. *Proceedings of the National Academy of Sciences* **112**, 7159-7164 (2015).

14 Melamed, D., Young, D. L., Gamble, C. E., Miller, C. R. & Fields, S. Deep mutational scanning of an RRM domain of the Saccharomyces cerevisiae poly (A)-binding protein. *Rna* **19**, 1537-1551 (2013).

15 Starita, L. M. *et al.* Activity-enhancing mutations in an E3 ubiquitin ligase identified by high-throughput mutagenesis. *Proceedings of the National Academy of Sciences* **110**, E1263-E1272 (2013).

16 Pokusaeva, V. O. *et al.* An experimental assay of the interactions of amino acids from orthologous sequences shaping a complex fitness landscape. *PLoS genetics* **15**, e1008079 (2019).

17 Sinai, S., Jain, N., Church, G. M. & Kelsic, E. D. Generative AAV capsid diversification by latent interpolation. *bioRxiv*, 2021.2004. 2016.440236 (2021).

18 Russ, W. P. *et al.* An evolution-based model for designing chorismate mutase enzymes. *Science* **369**, 440-445 (2020).
